# The Musicality of Non-Musicians: An Index for Assessing Musical Sophistication in the General Population

**DOI:** 10.1371/journal.pone.0089642

**Published:** 2014-02-26

**Authors:** Daniel Müllensiefen, Bruno Gingras, Jason Musil, Lauren Stewart

**Affiliations:** 1 Department of Psychology, Goldsmiths, University of London, London, United Kingdom; 2 Department of Cognitive Biology, University of Vienna, Vienna, Austria; UNLV, United States of America

## Abstract

Musical skills and expertise vary greatly in Western societies. Individuals can differ in their repertoire of musical behaviours as well as in the level of skill they display for any single musical behaviour. The types of musical behaviours we refer to here are broad, ranging from performance on an instrument and listening expertise, to the ability to employ music in functional settings or to communicate about music. In this paper, we first describe the concept of ‘musical sophistication’ which can be used to describe the multi-faceted nature of musical expertise. Next, we develop a novel measurement instrument, the Goldsmiths Musical Sophistication Index (Gold-MSI) to assess self-reported musical skills and behaviours on multiple dimensions in the general population using a large Internet sample (n = 147,636). Thirdly, we report results from several lab studies, demonstrating that the Gold-MSI possesses good psychometric properties, and that self-reported musical sophistication is associated with performance on two listening tasks. Finally, we identify occupation, occupational status, age, gender, and wealth as the main socio-demographic factors associated with musical sophistication. Results are discussed in terms of theoretical accounts of implicit and statistical music learning and with regard to social conditions of sophisticated musical engagement.

## Introduction

The ability to engage with music in sophisticated ways is a unique and universal human ability [1]. Participation in musical activities occurs in every known human culture [2]. However, the ways in which members of a society differentiate and specialise in their engagement with music varies greatly between cultures. Blacking [3] observed and described in detail how some cultures lack any notion of hierarchy according to musicianship status while others–particularly Western societies–make very clear distinctions between individuals, according to their ascribed specialist music skills and roles. This hierarchical notion of expertise in music persists in Western societies across almost all popular and art music styles and types of engagement. Success, excellence, and expertise can be ascribed to performing musicians, composers/song writers, music producers, recording engineers, DJs, music critics, music academics and avid music ‘connoisseurs’ alike.

However, as Levitin [4] recently argued, almost all of the scientific instruments used to study musicality and musical achievements in Western society are centred on the ability to play an instrument and the expertise of performing musicians in Western art music, ignoring the skills necessary for successfully engaging with music in other ways besides playing an instrument. The recent works of Hallam [5], as well as Hallam and Prince [6], suggest a more multifaceted and nuanced view of musicality that is broader than that typically assessed via traditional tests, which includes musical understanding, appreciation, evaluation, and communication alongside playing an instrument, improvisation and having a good sense of pitch and rhythm. However, to date no measurement tool has been created following these lines of thought.

This paper describes the development and evaluation of the Goldsmiths Musical Sophistication Index (Gold-MSI), a novel instrument that measures musical sophistication in a comprehensive way by explicitly considering a wide range of facets of musical expertise as they occur in a Western society. The instrument is designed to measure the broad range of individual differences in the general population, while placing less importance on the much smaller pathological groups (e.g. ‘amusics’ [7], [8]) and highly specialist populations (professional musicians). Data from 147,633 individuals, who took both the self-report inventory as well as the battery of listening tests from the Gold-MSI, are presented. Relating self-reported musical behaviour to the performance on the listening tests enables us to determine the extent to which skill acquisition and expertise may be related to reported patterns of musical engagement. Since many musical skills are not explicitly trained, but are developed through repeated and focused engagement with music, the results from this large sample highlight the processes of implicit learning that take place during enculturation with Western music. Finally, using socio-economic data from 90,474 British participants in our sample, we describe the typical conditions under which musical sophistication develops, and discuss mechanisms by which individuals can continue to engage with music at a high level throughout the lifespan.

### Assessing Musical Abilities and Musical Behaviour

The assessment of musical abilities and achievements has a relatively long scientific history. Seashore published the initial version of his test of ‘musical talents’ in 1919 and since then a number of tests [9]–[14] have been developed to assess musical abilities, the potential to develop musical skills, or musical achievements. Boyle and Radocy [15] provide a systematic summary of musical aptitude tests published over the course of the 20^th^ century and describe how most of them were intentionally designed for specific purposes in (Western) music education. In addition to this long tradition of musical aptitude tests primarily designed for use in music education, a number of listening tests have been developed more recently with a focus on academic research and suitability for an adult population. Gordon’s Advanced Measures of Audiation [10] comprise a “same” versus “different” comparison task for pairs of newly created single line melodies with increasing complexity and length where participants also have to indicate whether the difference between two melodies is rhythmical or tonal in nature and the respective responses give rise to a rhythmic and a tonal test score. The Musical Ear Test [12] employs a very similar experimental paradigm and stimuli, but tests melody and rhythm perception with two different subtests. The Montreal Battery for the Evaluation of Amusia [7] also makes use of the same-different comparison paradigm with short melodic or rhythmic single line sequences as stimuli across 4 of its 5 subtests where tests differ in the quality of change that can occur between the paired items. The most recent musical perception battery is the Profile of Music Perception Skills (PROMS) [14] which also makes exclusive use of the same-different comparison paradigm for its 9 subtests. However, the stimulus material of the PROMS extends beyond pairs of melodic and rhythmic single line sequences and also includes samples of instrumental tones and sinusoids as well as multi-layer sequences. It is worth noting that none of these test batteries includes a self-report inventory. Furthermore, almost all of the aforementioned batteries use artificially created experimental stimuli. This strategy is helpful in order to control for familiarity effects but bears the risk of producing musical stimuli of little ecological validity and little resemblance to real music, and which are fairly remote from the participants’ musical experiences and listening expertise and therefore can advantage participants who have learned to engage with more abstract musical material, e.g. through instrumental lessons or ear training. The educational perspective of most of the earlier musical aptitude tests explains the similarity to ear-training exercises, such as those used in Western art music education where individual elements of musical structure (most often melody, rhythm, or harmony) are commonly presented in isolation and assessment tasks often seem artificial compared to most people’s real-world listening behaviours. Perhaps unsurprisingly, therefore, individuals with formal training in Western art music typically achieve higher scores on these tests. However, these traditional tests of musical achievement overlook a variety of musical achievements or skills [16], such as the abilities to verbally communicate about music at a high level, to use music effectively to manipulate one’s own emotional states and those of others, and to compare music stylistically. Many of these skills form the basis of musical professions such as DJing, music journalism, or music production.

Similarly, items in traditional musical aptitude tests are often taken from or created in the style of simple pieces of Western art music or the folk song repertoire, and in almost all cases ignore multi-instrument textures and sound quality as relevant dimensions in many Western music styles. For instance, neither musical sound or timbre, nor musical excerpts with several instruments playing together feature in either of the two most commonly used musicality tests [10]–[11]. Seashore [17] justifies a focus on more simple musical stimuli by arguing that the correct processing of structural musical ‘atoms’ is a pre-condition for the successful decoding of more complex musical contents and hence testing the ability to process musical ‘atoms’ would be a valid proxy for indexing higher musical skills (see pp. 3–4 [14] for a similar argument regarding the basic and abstract sound patterns predominately employed as stimuli in PROMS). This argument probably holds true for predicting achievements in traditional Western music education but is perhaps less relevant for the skilled engagement with music in other forms and for expertise with other types of Western music.

Indeed, one motivation for the development of the Gold-MSI inventory and test battery was to devise tasks for assessing musical skills that are more akin to real-world skilled listening behaviours and that would incorporate stimulus items from a wider range of musical styles.

Compared with the long history of musical aptitude tests, most self-report questionnaires for the assessment of musical behaviour are relatively recent [18]–[22]. However, to our knowledge, none of these self-report instruments focus on the expertise or the differentiation of skilled musical behaviours, aside from formal musical training. Hence, one of the main goals of the Gold-MSI project was to develop a self-assessment instrument that can measure expertise with regard to a variety of musical activities, not only instrumental expertise. The combination of a self-assessment instrument and high-level musical listening tests that include complex musical material and employ different testing paradigms is the second main goal of this study, which distinguishes the Gold-MSI from existing musical test batteries and makes it a research tool that complements the musical ability tests referenced above.

### Defining Musical Sophistication

In line with Ollen [21], we deliberately adopt ‘musical sophistication’ as a term that has been used infrequently in earlier research and is therefore less loaded with biases and preconceptions than more commonly used terms such as musicality, musical talent, ability, aptitude, or musical potential (see [15], [23], [24] for discussion of different terms and concepts). In our conceptualisation, musical sophistication is a psychometric construct that can refer to musical skills, expertise, achievements, and related behaviours across a range of facets that are measured on different subscales. We assume that multiple facets of musical sophistication can develop through active engagement with music in its many different forms and that individuals vary in their level of sophistication on these different facets (see [25] for the close relationship between ecological validity and multi-dimensionality of musical aptitude tests). We posit that high levels of musical sophistication are generally characterised by a) higher frequencies of exerting musical skills or behaviours, b) greater ease, accuracy or effect of musical behaviours when executed, and c) a greater and more varied repertoire of musical behaviour patterns. This means that highly musically sophisticated individuals are able to respond to a greater range of musical situations, are more flexible in their responses, and possess more effective means of achieving their goals when engaging with music. Note that this definition of musical sophistication is sufficiently abstract to apply equally to performing musicians of all styles as well as to music writers and commentators, and to individuals who apply music in functional ways such as DJs, music educators, producers, or music engineers. We further assume that differences in observable behaviour are related to levels of differentiation in categorising and processing music in the cognitive system of individuals. In line with expertise research literature from other domains [26]–[32], we assume that, with greater expertise, the representational cognitive system for a domain will differ in its level of sophistication, i.e. cognitive representations will be more structured, and will exhibit a clearer hierarchical organization as explained and defined by e.g. Ericsson and Smith [33], Glaser [34], and Honeck, Firment, and Case [35]. However, this definition makes no assumption with regard to how musical sophistication is acquired and whether it mainly stems from natural talent [36], genetic predispositions [37]–[40], or is largely a result of learning processes.

Our definition of musical sophistication builds on concepts that are similar to those introduced by Hallam and Prince [6], and Ollen [21] who also stressed the multi-dimensional nature of musical sophistication, including aural skills, receptive responses, and the different abilities to make music [21]. However, our conceptualisation and implementation of musical sophistication differs from these earlier characterisations in that it emphasises other skilled musical behaviours besides instrumental practice, is not biased towards art music, includes a self-assessment of musical skills, models musical sophistication as a continuous parameter, and is explicitly linked to cognitive theories of expertise in other domains.

### Musical Skills in ‘Non-musicians’

Much previous music-related research has been preoccupied with measuring behavioural, cognitive, and brain structural/functional differences between musicians and non-musicians, where the criteria used to define these groups have mostly emphasised musical abilities conferred by musical training, including variations in terms of the criteria used to establish the groups of interest [41]–[46]. But the emphasis on formal musical training (on an instrument, including voice) has likely overlooked the possible effects of a type of expertise that does not involve theoretical or technical knowledge of music, and can be present in people who consider themselves non-musicians. Studies published over the past decade have suggested that music listening expertise does not need to be taught; in fact, the knowledge gained through formal musical training may be rather tangential to the skills required to be an expert listener. Young infants, before they have had the opportunity to receive formalised training, demonstrate sophisticated musical abilities, including the ability to distinguish intervals, recognise folk songs [47], and detect metrical deviations in music from their own musical culture as well as from a non-native one [48]. Thus, as with speech [49]–[50], musical enculturation shapes perceptual capacities via exposure. Implicit learning of this sort relies upon the brain’s ability to internalise statistical regularities from its exposure to auditory stimuli [51]–[56]. The fact that implicit learning takes place incidentally, without awareness, and can rarely be verbalised tends to result in a general underestimation of the musical abilities of people without formal training. Nevertheless, there is clear evidence that these individuals can possess considerable implicit knowledge of musical structure across a range of different tasks (see overviews provided by [57]–[59] and the related notion of ‘musical sleepers’ [14]), and that differences in musical listening patterns can also affect non-musical abilities [60].

The fact that knowledge of musical regularities and structure can be gained implicitly does not entail that the exposure to music is necessarily ‘passive’. Although it may seem effortless, listening to music is an active process, engaging the listener in a process of parsing, segmenting, and encoding a complex stream of auditory events, and extracting structure at multiple hierarchical levels, requiring concerted neural activity across auditory association areas in the temporal lobes, auditory working memory areas in the frontal lobes, and emotional centres in the limbic system [61]–[62]. Recent work has stressed the extent to which certain aspects of musical listening can result in top-down interactions from cortical to subcortical areas, in order to better encode the most relevant features of the incoming stimulus [63]. Clearly, the ways in which individuals actively engage with music can vary, and are related to many factors including the amount of focused listening per day, the importance attached to music in everyday life, the extent to which an individual responds emotionally to music, and the degree to which an individual takes part in music in informal ways (e.g. singing along to tunes, exchanging views on music with others). Hence, a major goal of the present study is to provide a standardised measurement instrument to examine musical sophistication, which will allow future studies to examine how differences across this profile (or in facets of it) may relate to differences in perceptual, cognitive, neurological, or even immune system function.

### Overview of Studies

This paper comprises five studies relating to the development and refinement of the Gold-MSI, a comparison of objective and self-reported assessments of musical sophistication, and finally an analysis of the socio-demographic correlates of musical sophistication in a large sample of British participants. Study 1 reports the development of the Gold-MSI self-report inventory on a large data sample gathered through an online survey with BBC Lab UK. Study 2 uses a different sample (from the same survey) to confirm the measurement structure of the self-report instrument and reports the structural relationships between different facets of musical sophistication using a confirmatory approach. Study 3 reports psychometric indicators of internal reliability, and external convergent and discriminant validity of the self-report inventory as well as correlations with a standard personality inventory. Study 4 compares the results from the self-reported facets of musical sophistication with results from two musical listening tasks from the Gold-MSI battery, and investigates how self-reported musical behaviour and objectively measurable listening abilities are related. Finally, Study 5 explores the socio-economic conditions of musical sophistication by relating scores from self-report inventory and listening tests to variables of socio-economic status, such as education level, occupational status, and wealth. The Ethics Board of Goldsmiths, University of London approved the research undertaken and reported in the manuscript.

## Study 1: Developing a Self-Report Inventory for Musical Sophistication

The development of the self-report inventory was based on a systematic review of the existing literature described above, covering questionnaire instruments of musical behaviour [18]–[22], [60], tests of musical abilities [9]–[15], and inventories for assessing expertise in other domains (e.g. physics [27]; wine [28]–[29]; computer programming [31]; badminton [32]). The objective of the review was the development of a new self-report inventory measuring the most common forms of skilled musical behaviour in the general Western population by deriving sub-scales for different facets of ‘musical sophistication’. On the basis of the literature review as well as the conceptual definition of musical sophistication given above we initially posited five distinct hypothetical dimensions of musical sophistication merely to provide conceptual guidance at the item writing stage, namely resource allocation to music, music making, functional use of music, ability to verbalise musical experiences, and perceptual-cognitive skills.

The five hypothetical dimensions served to orient the writing of the initial pool of inventory items in the form of statements that could be endorsed to varying degrees on a rating scale. In item writing we ensured as much as possible that, within each dimension, positively and negatively phrased statements were balanced, that statements would apply to any musical style and any age group, and that as many potential behaviours of interest as possible would be covered for each dimension. The target population for responding to the items was composed of adults with a range of levels of formal musical training (from no training up to professional level), and we calibrated the items towards the level of musical behaviour and abilities that could be expected in the general population by using appropriate adverbs (e.g. ‘mostly’, ‘rarely’, ‘never’, ‘always’). We did not try to capture finer grained differences between high-level or professional musicians. The first iteration of the inventory comprised 153 statements written independently by three of the authors (DM, BG, LS). Each item was then jointly scrutinised and ambiguous items, quasi-synonymous items, items that did not fit with the overall concept of musical sophistication, and items that would potentially apply to only a small subpopulation were eliminated from the item pool. The remaining 111 items were then used in a pilot survey. For each of the five hypothetical dimensions we ensured that roughly equal proportions of items were stated positively. We adopted the same seven-point scale for all items ranging from complete agreement to complete disagreement. This scale includes a middle (i.e. neutral) category and represents a compromise between an interval scale providing data for subsequent parametric analyses and a manageable number of categories where each category retains a meaning that can be expressed verbally.

A pilot survey using an online questionnaire with these 111 items was launched via the BBC’s main Science webpage [64] for one week. This yielded responses from 488 participants from a broad age range. The data of the pilot survey were then subjected to a series of factor analytic techniques. In addition, we employed individual item analyses using classical test theory as well as item response models to reduce the pool of items. The analytic steps of this process are analogous to how the item reduction was carried out on the actual dataset of Study 1 reported in the results section below. In addition, the details of the analysis of the pilot data are given in Textual Description S1 and in a publicly available technical report [65]. Eventually, this pilot data gave rise to a solution comprising 70 items on 7 factors and explaining 53.6% of the variance, with the 7 subscales having very good psychometric properties (values of Cronbach’s alpha ranging between .693 and .921).

### Method

The 70-item self-report inventory was launched in January 2011 as part of the online test battery *How Musical Are You?* [66], developed by BBC Lab UK and promoted across the BBC broadcast network. 148,037 participants completed the self-report inventory as part of the test battery in 2011. From this sample we excluded individuals who mainly chose the same response category across the 70 (unreversed) items (i.e. variance <2 *SDs* below mean variance). This excluded 404 participants and left 147,633 in the sample. In order not to overfit the data, and to obtain unbiased estimates of model fit, we split the full sample into a training dataset (n = 73,894) used for the development of the inventory reported in Study 1, and a test dataset (n = 73,739) used for the confirmatory analysis in Study 2.

#### Participants

45.2% of the participants from the training sample were female and 54.7% were male. Mean age was 35.2 years (*SD = *15). Participants were mainly UK residents (66.9%) but because the *How Musical Are You?* test battery was an open online application, the sample also included participants from other, albeit mainly Western and English-speaking, countries (most frequently named: USA: 14.2%, Canada: 2.3%, Australia: 1.1%). The ethnic background of the participants was mostly white (84.1%) but also included a wide range of participants from non-white backgrounds (most frequent: Asian/Indian/Pakistani/Bangladeshi: 3.4%; Mixed Race: 2.3%, East/South-East Asian: 1.8%). The sample contained a large spread in terms of education (undergraduate degree/professional qualification: 34.1%, still in education: 23.4%, postgraduate degree: 19%, second school degree around 18 years (e.g. British A-levels): 11.8%, first school degree around 16 years (e.g. British GCSE/O-levels): 7.5%, etc.) as well as in terms of the current profession of the participants (Other: 19.4%, Education/Training: 12.4%, Unemployed: 10.7%, Information technology: 7.1%, etc.). Only 1.8% stated ‘Music’ as their occupation. There was no incentive for the participants other than the individual feedback that was based on the data norms derived from the pilot.

#### Procedure

Participants were required to obtain an online-identifier from the BBC (the BBC-ID) and then log into the actual test battery. They completed the self-report inventory along with a short demographic questionnaire and four tests of musical ability. If taken without pauses, the entire testing procedure took about 25 minutes. Participants were then given online feedback on their ‘relationship with music’ in the form of the percentile of their scores as well as short interpretations of the numerical score. In addition, participants were given the results of the four musical ability tests, and debriefing information about the online study itself. Participants were only able to take the test once with the same BBC-ID. However, it was technically possible for an individual to create a second BBC-ID and to re-take the entire test and we therefore included a question to identify a small number of re-takers (0.02% of the full sample) which were left in the data sample. The data were fully anonymised before analysis and the research team did not have access to information that could lead to personal identification, such as email or IP addresses.

### Results and Discussion

#### Identifying the factor structure of the self-report inventory

Identifying the dimensionality of the data in factor analysis is crucial, especially if the aim of the analysis is to develop a multi-dimensional measure with corresponding sub-scales. We therefore looked at the convergence of different criteria for deciding on the appropriate number of dimensions. We used different factor extraction methods (maximum likelihood factor analysis, principal axis factoring using an iterative least squares optimisation, minimum residual factor analysis) and as criteria employed the screeplot [67], Kaiser’s criterion of eigenvalues >1, parallel analysis on random and resampled datasets of the same size [68]–[69], Velicer’s Minimum Average Partial (MAP) criterion [70], and Revelle and Rocklin’s Very Simple Structure (VSS) criterion [71] (all analyses were carried out using the R software environment and the R package psych [72]). Initially, we did not find any convergence for the different methods, obtaining indications for optimal solutions ranging from 1 to 16 factors. One potential reason for the disagreement of the different criteria can be the presence of a strong general factor that can eclipse less strong group factors. When we investigated this possibility we found that the Very Simple Structure Criterion for a solution with complexity level 1, as well as the ratio of the eigenvalue of first factor to the number of variables (0.298; [73]), indeed suggested that a general factor (second-order factor) might be present in the data, accounting for the correlations between the first-order group factors. We tested for the presence of a general hierarchical factor using McDonald’s coefficient omega [74] which has been shown to be the most sensitive and the most reliable measure for testing for the presence of a hierarchical factor [75]–[76]. For all hierarchical factor solutions (based on maximum likelihood factor analysis) with one general factor and 3 to 16 group factors we obtained values of omega ranging from 0.721 to 0.834, giving clear evidence of a general factor of musical sophistication (regardless of the true number of group factors). However, the absolute fit indices for a simple model having only a general factor and no group factors indicated only a mediocre fit (RMSEA = .079, Tucker-Lewis Index =  .589, Bentler CFI =  .601). Adding group factors into the model increased the absolute, as well as the comparative fit, as measured by the BIC (range from 1040.747 to 139.912 for 1- to 16-factor solutions), clearly suggesting that group factors are necessary in addition to the general factor to account for the data.

In order to discount this strong general factor in the search for the correct number of dimensions, we performed a 1-factor maximum likelihood factor analysis and extracted the matrix of residuals for a subsequent analysis of the dimensionality of the data using the same criteria as above. For all extraction and rotation methods employed on this input matrix, the MAP criterion always indicated 6 dimensions to be optimal. In addition, the 6^th^ factor received an eigenvalue of 0.99 in principal axis, maximum likelihood, and minimum residual factoring and the VSS criterion indicated for most extraction methods (using oblique rotation) and most complexity levels that a solution with 6 factors was optimal. We interpreted this as a clear indication of the presence of 6 group factors in addition to a hierarchical general factor in our data.

On the training dataset we fitted a model with a hierarchical factor and 6 group factors using maximum likelihood extraction, oblimin rotation and the Schmid-Leiman procedure [77] to extract the general factor from the inter-correlations of the group factors. The model had a high value of omega (.74) and a very good overall data fit (RMSEA = .046, TLI = .858). The eigenvalue of the general factor was 16.2 and the 6 group factors had eigenvalues in the range from 4.5 to 1.7.

In order to obtain a simple factorial structure, and to construct non-ambiguous subscales of musical sophistication, we fitted a variant of this model as a structural equation model with a general factor, 6 group factors, and where each of the 70 items was only related to the one group factor where the loading was highest. This model still possessed a very good absolute fit (χ^2^ = 473746, df = 2275, RMSEA = 0.053, TLI = .813, CFI = .823) with the general factor having an eigenvalue of 19.2 and the 6 group factors ranging between 2.9 and 1.2. We accepted this simple model as a good enough fit to our data to use it as a starting point for the subsequent refinement of the subscales. It is important to note that the construction of the six-plus-one factor model does not represent a ‘natural’ or ‘true’ model of musical sophistication but is partially due to our theory-driven approach that was also informed by evidence from prior literature. A less theoretical approach might have yielded a different set of dimensions, both in kind and in number.

#### Refinement of subscales

We first inspected each of the 6 factors in terms of their content, their psychometric properties, and their compatibility with the general concept of musical sophistication. All items except two loaded positively on a single factor. The two negatively loading items were excluded from all further analyses. This initial version of the 6 subscales comprised between 7 and 20 items per subscale with values of Cronbach’s alpha ranging between .803 and .918.

Factor 1 comprised 20 items that covered a range of active musical engagement behaviours (e.g. “I keep track of new music that I come across”, “I often read or search the internet for things related to music”) as well as the deliberate allocation of time and money on musical activities (e.g. “I don’t spend much of my disposable income on music”, “I listen attentively to music for _ hours per day”). We therefore named this factor *Active Engagement*.

Factor 2 had 15 items, each representing the self-assessment of a cognitive musical ability, and most of them related to music listening skills (e.g. “I can compare and discuss differences between two performances or versions of a musical piece”, “I can tell when people sing or play out of tune”). We termed this factor *Perceptual Abilities*.

The 11 items of Factor 3 combined questions about the extent of musical training and practice (e.g. “I engaged in regular daily practice of a musical instrument including voice for __ years”, “At the peak of my interest I practised on my primary instrument including voice for __ hours per day”), and about the degree of self-assessed musicianship (“I would not consider myself a musician”, “I have never been complimented for my talents as a musical performer”). We termed this factor *Musical Training*.

Factor 4 consisted of seven items that reflected different skills and activities related to singing (e.g. “After hearing a new song two or three times I can usually sing it by myself”, “I am not able to sing in harmony when somebody is singing a familiar tune”) and was termed *Singing Abilities*.

Factor 5 had eight items describing reactive behaviours that are generally carried out in response to an external music source, and where subjects do not plan or seek out the behaviour in advance (e.g. “I hardly ever hum or sing along to music”, “I rarely tap or clap along when listening to music”, “When I hear a catchy tune I find myself moving to the beat”). Unlike the items of the other factors (e.g. Factor 2, *Perceptual Abilities*), the items of this factor did not suggest that behaviours could become more skilful or varied or sophisticated, rather that they happen more frequently. This is in line with the notion that we do not regard people to be more musically sophisticated merely when they find themselves tapping to music more frequently, but also when their tapping is more precise, accurate, or executed along with more complex stimuli. Also, the behaviours described by the items on Factor 5 all expressed rather reactive behaviours in response to incidental music listening rather than goal-directed active engagement with music, which seemed to go against the general idea of musical sophistication as a repertoire of skilled and adaptive behaviours that develop through active involvement with music. This view was supported by the fact that Factor 5 had a substantially lower association with the General Musical Sophistication factor than any other factor in the hierarchical structural equation model (parameter estimate_factor 5_ = 1.03; mean estimate_other factors_ = 1.48, CI_95%_ = 1.09; 1.86). Thus, the items of this factor seemed to have little content validity and this factor was statistically less associated with the general factor. We therefore decided to discard Factor 5 and the items associated with it during the subsequent development of the self-report inventory.

Factor 6 had nine items associated with it that covered different and mainly active behaviours related to emotional responses to music (e.g. “I am able to talk about the emotions that a piece of music evokes in me”, “I sometimes choose music that can trigger shivers down my spine”). We termed this factor *Emotions*.

It is worth noting that Factors 1 (active engagement) and 3 (musical training) mainly comprise items that describe past or current music-related behaviour while Factor 2 (perceptual abilities), Factor 4 (singing abilities), and the new Factor 5 (emotions) mainly contain items where different aspects of a musical skill are self-assessed. Combining these two qualitatively different types of factor/items provides an assessment of musical sophistication that includes a quantifiable record of relevant behaviours potentially leading to the acquisition and refinement of skills, as well as subjective judgements regarding the skill level attained.

The goal of the refinement of the subscales was to reduce the number of items while retaining the good psychometric properties of each subscale. The item response theory approach [78]–[79] was used to reduce the number of items per subscale. We fitted constrained and unconstrained graded response models (GRM) [80] to the items of each subscale using the training dataset (we used the R-package *ltm* for the GRM [81]). In all cases, the unconstrained model (which lets the discrimination parameter vary across items) fitted the subscale data significantly better (*p*<.001). For the unconstrained GRM of each subscale, we inspected the plots of the item information curves, the total test information value, the individual item information values, the item discrimination parameters, and the distribution of overall scores for the subscale. With the aim of reducing the number of items per subscale, we carried out the following analytical steps: a) a subset of items that covered the full range of the latent ability scale having high item information values were identified, b) items that contributed little in terms of the overall test information were excluded, and c) the item with the highest item information value was selected where items were overlapping in content. Following this procedure we arrived at considerably shorter scales that comprised between 6 and 9 items but maintained similar values of Cronbach’s alpha as an indicator of their reliabilities (ranging between .789 and .900, see Table S1 in File S1 for the assignment of the 38 items to the five sub-scales).

In summary, the exploratory analysis presented in Study 1 identified a strong general factor of musical sophistication as well as 6 group factors, 5 of which were clearly compatible with the initial definition and overall notion of musical sophistication that arose from a comprehensive examination of the relevant literature. With the help of item response analysis, we were able to reduce the number of items in order to form shorter subscales. Despite the reduction in items, we were able to achieve good levels of reliability across all subscales. Study 2 used the test dataset (which had not been used to derive the factor structure of the inventory) to investigate whether the scale and subscale model would still achieve an acceptable model fit on a different set of data using a confirmatory approach.

## Study 2: Assessing the Adequacy of the New Self-Report Inventory

The purpose of this study was to assess, on a new dataset, the unbiased fit of the reduced self-report inventory that was developed on the training dataset. In addition, we also tested the hypothesis that there was indeed a strong general factor of musical sophistication or, alternatively, that the data could be equally well accounted for by a simpler factor structure, not taking the relationships between factors into account. Therefore, we specified four models that differed both in complexity, and also in whether and how inter-factor correlations were accounted for.

### Method

#### Participants

The test dataset was used for this analysis. This dataset comprised 73,739 individuals of which 45.2% were female. The distributions of the countries of residence as well as the education levels and professions were highly similar to those reported above for the training dataset (the differences were in the order of 0.1%) and details are therefore not reported here.

#### Procedure

The procedure was identical to that used in Study 1.

### Results and Discussion

We specified four models differing in their factor structure: Model 1 was specified as a hierarchical model where a general factor was hypothesised to impact on the five group level factors, which in turn were suggested to impact on the individual items associated with them. In terms of model complexity, this model requires 81 free parameters. Model 2 was the Schmid-Leiman transformed variant of the hierarchical model where the general factor is partialled out from the group factors and impacts directly on the 38 items in addition to the influence of the individual group factors. This model has 114 free parameters to estimate. Model 3 is a simple confirmatory factor analysis model without a general factor and where only the relations between group factors and items are modelled and therefore only 76 free parameters are required. Model 4 is similar to the non-hierarchical Model 3 but allows for factor inter-correlations between the five group factors and needs to estimate 86 parameters.

The χ^2^ values of all models showed a highly significant departure from an exact fit, which is not surprising given the large sample size. Because the four models are not nested into each other, we used the Bayesian Information Criterion (BIC) to compare models which gave rise to the following order (from best to worst) Model 2> Model 4> Model 1> Model 3.

Thus, in line with the high values of McDonald’s coefficient omega obtained on the training dataset in Study 1, we found that the three models that take into account the inter-factor relationships, either in the form of individual inter-factor correlations (see Table S2 in File S1) or modelled as a general factor, fitted the data significantly better than Model 3, which assumes independence between factors.

The approximate fit indices for Models 1, 2, and 4 indicate a reasonably good fit to the data in absolute terms. In addition, all parameters (regression coefficients, co-variances and variances) in Models 1, 2, and 4 were highly significant and in no instance did the value of a parameter’s standard error exceed the threshold of 1/n^1/2^ as suggested by McDonald ([74] p.187). The fact that Model 2 (the Schmid-Leiman variant) fitted the data best suggests that its additional free parameters are justified to explain the structure in the data.

**Figure 1:**
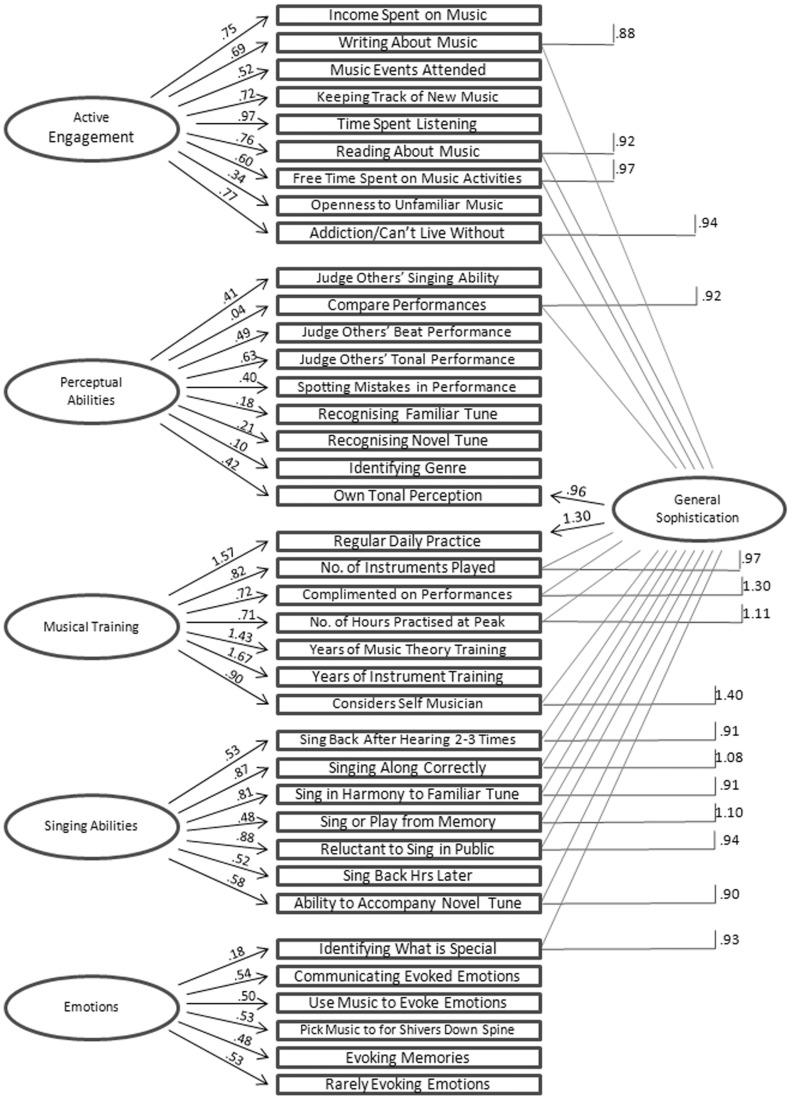
Factor structure of reduced self-report inventory as formalised by model 2, the Schmid-Leiman variant of the confirmatory factor model.

However, for the practical purposes of the development of a new inventory of musical sophistication, the difference in fit between Models 1, 2, and 4 has no consequences, except for the construction of a general scale of musical sophistication indexing the general factor. To this end we inspected the distribution of regression coefficients ordered by coefficient size from the 38 items onto the general factor in Model 2. The ordered distribution, which has a format similar to a screeplot, has several discontinuities and we decided to include items above a break in the plot that splits the number of items approximately in half. This led us to select 18 items with a coefficient above 0.88 to index musical sophistication in general. These 18 items were drawn from all five subscales but there was a clear preponderance of items from the Musical Training and the Singing Abilities subscales. The factor structure and regression coefficients of Model 2 are given in Figure 1.

In summary, the results of Study 2 confirmed that the structural and measurement models of the self-report inventory developed in Study 1 hold true on an evaluation dataset and that our data on musical sophistication are best modelled using a general factor as well as 5 group factors to index different facets of musically sophisticated behaviour.

## Study 3: Reliability, Validity and Correlates of the Self-Report Inventory

Studies 1 and 2 developed and confirmed the adequacy of the item and factor structure of a new self-report inventory for musical sophistication. In Study 3, we looked at the reliability and validity of the new inventory in comparison with other music-related self-report scales as well as how it correlated with two short personality inventories [82]–[83], following suggestions that musical behaviour and engagement may be linked to certain dimensions of personality [84].

However, as with self-report inventories for skills and abilities in other domains, it cannot be taken for granted that self-assessed and actual skill levels correlate highly, or indeed that they converge at all. For example, Paulhus, Lysy, and Yik [85] found only low correlations (<.3) between several self-report measures of intelligence and scores on an intelligence test. However, Furnham’s test of self-assessed multiple intelligences [86] achieved correlations of at least between.3 and.5 with standard intelligence tests [87]. Reasons for the low correlations between self-assessed and actual levels of skill can be related to differences in the psychometric constructs compared, low levels of self-awareness in the particular domain, or an inappropriate frame of comparison for the self-assessment, as well as biases potentially introduced by social desirability, extreme levels of self-confidence, or other personality traits. Hence Study 3 also compares scores from the Gold-MSI self-report inventory with the performance on Gordon’s Advanced Measures of Audiation (AMMA) [10], an established musical aptitude test that is widely used for evaluation and prediction of achievements in Western music education [88]–[93], as well as in behavioural [94]–[95] and neuroscientific research [96]–[97].

Because we used different samples of participants we subdivided Study 3 into four sub-studies. Study 3a made use of the full (“BBC”) sample to derive data norms, as well as indicators of internal reliability for the five subscales and the general factor. Study 3b reports on the test-retest reliability of the self-report inventory over two different time intervals, as well as on correlations with the AMMA listening tests to assess external validity.

Study 3c also investigated the convergent validity of the self-report inventory by looking at correlations with the Musical Engagement Questionnaire (MEQ) [22]. The MEQ was designed as a ‘broad-based questionnaire measuring the experience of music’ and assessing the spectrum of psychological facets of musical experiences (p. 331). The MEQ is not primarily concerned with the degree of sophistication of musical behaviours. However, the MEQ consists of six subscales, two of which can be hypothesised to measure constructs related to subscales of the Gold-MSI self-report inventory while we expected lower correlations for the other subscales.

Finally, Study 3d explored the relationships between the six sub-scales of musical sophistication and two standard personality inventories: the Ten-Item Personality Inventory (TIPI) [83] measuring the ‘Big Five’ personality traits as well as Eysenck’s [82] 12-item extraversion scale. A number of studies in the past have found clear correlations between personality traits and measures of musical behaviour [98]–[105]. Among the ‘Big Five’ personality traits, openness to experience has been suggested to be closely linked to musical engagement. Openness to experience can be understood as the desire to broaden the range of experience in a lifetime and individuals scoring high on this construct tend to have a good awareness of the arts [98]. Ample support has linked openness to experience with aesthetic interest in general [99]–[100] and to music in particular. With regards to music, links with openness to experience have been discovered with the appreciation of unfamiliar music [101], musical preferences [84], [102], musical listening styles [103], self-assessed musical intelligence [86], and more diverse music tastes [104]. In addition to this greater general engagement with the arts and music, openness to experience has also been suggested to correlate with greater emotional appreciation for aesthetic stimuli and music in particular. Vuoskoski and Eerola [105] correlated ‘Big Five’ personality factors with the intensity of felt emotions in response to music and found that people scoring highly on openness to experience were more likely to experience the most powerful emotional reactions when listening to sad-sounding and gentle music. In addition to these strong links between music and openness to experience in the general population, there has been a considerable number of studies investigating the personality structure of accomplished performers. Several hypotheses have been put forward within this research strand, including the stereotype of the ‘bold introvert’ [106] (for a critique see [107]), and personality differences between players of different instrumental groups, such as string players, brass players, or singers [108]–[112]. However, possibly due to the lack of a valid and reliable measurement instrument, the relationship between personality and musical abilities in the general population has generally been overlooked so far.

### Method

#### Participants

For Study 3a we combined the data training- and test sets. This dataset comprised 147,633 participants, of which 45.2% were females. The distributions of the countries of residence as well as the education levels and professions only differed from the training dataset reported above in the order of 0.1% and are therefore not reported here.

For Study 3b, 53 participants took the self-report inventory twice in a controlled lab environment in two testing sessions that were scheduled 64 days apart on average (minimum of two weeks) to minimise memory effects. 44 of these participants were also tested on the AMMA musical listening test. Participants were mainly university students from Goldsmiths, University of London, as well as other higher education institutions in London. Of the 53 participants, 52.8% were males and mean age was 26.3 years (*SD = *9.6).

For Study 3c the MEQ and the Gold-MSI were administered to 141 participants who were recruited from the Goldsmiths undergraduate community and tested in a classroom environment in the summer and autumn of 2011. 73% were female and mean age was 21.3 years (*SD = *5.9).

Study 3d used the data from 224 participants who were assessed with a paper version of the TIPI personality inventory [83] as well as the musical sophistication self-report inventory. About half of the participants were undergraduate students at Goldsmiths while the other half were young adults from the London area. 73.2% were females and mean age was 24.6 years (*SD = *11.4). Several studies [113]–[116] found that Introversion-Extraversion correlates with aspects of musical behaviour but results with respect to the direction of the correlation are ambiguous. We therefore also included Eysenck’s [82] more comprehensive Extraversion scale in addition to the 2-item extraversion short scale as part of the TIPI.

#### Procedure

The procedure of Study 3a was identical to that described in Study 1. In Study 3b the self-report inventory was administered on screen and in a controlled lab environment. As part of the two testing sessions, participants were tested on the AMMA musicality test as well as a range of other measures of cognitive ability (not reported here). Participants were remunerated with £20 for their participation after the second session.

For studies 3c and 3d participants were administered a paper version of the different self-report inventories and were not remunerated.

### Results and Discussion

#### Study 3a

We calculated three different (but related) measures of internal reliability (Cronbach’s alpha, McDonald’s omega total [76], and Guttman’s lambda6 [117]) for the five subscales and the general musical sophistication scale. As Table 1 shows, all scales possess good or very good estimates of internal reliability. Thus, in terms of reliability the five subscales as well as the scale for general sophistication seem to be suitable for testing individual differences.

**Table 1:** The fit statistics of the four structural equation models confirming the factor structure of the self-report inventory on the data test set (n = 73,739).

	Model 1	Model 2	Model 3	Model 4
	(Hierarchical)	(Schmid-Leiman)	(Simple Factor)	(Factor Inter-Correlations)
?^2^	215093	166170	382428	196363
df	660	627	665	665
BIC	216001	167448	383279	197326
TLI	.841	.874	.718	.853
CFI	.850	.884	.734	.863
RMSEA	.066	.060	.088	.064
SPMR	.068	.064	.276	.059

Footnote. BIC = Bayesian Information Criterion, TLI = Tucker-Lewis Index, CFI = Bentler’s Comparative Fit Index, RMSEA = Root Mean Square Error of Approximation, SPMR = Standardized Root Mean Square Residual.

Table 2 also gives the ranges, means, and standard deviations of the data norms derived from the subscale raw scores (using unit weighting of the items) for all five subscales as well as the general musical sophistication scale. The full data norms including all percentile scores are given in Table S3 in File S1.

**Table 2:** Summary statistics and indicators of reliability for Gold-MSI subscales and general musical sophistication factor (n = 147,633).

	Active Engagement	Perceptual Abilities	Musical Training	Singing Abilities	Emotions	General Sophistication
Mean (SD)	41.52 (10.36)	50.20 (7.86)	26.52 (11.44)	31.67 (8.72)	34.66 (5.04)	81.58 (20.62)
Scale Maximum	63	63	49	49	42	126
Scale Minimum	9	9	7	7	6	18
alpha	.872	.873	.903	.870	.791	.926
omega.tot	.874	.874	.904	.871	.792	.927
G6	.864	.867	.905	.866	.768	.938

#### Study 3b

All test-retest correlations for the five subscales and the general factor were found to be very high (between.857 and.972) and significant as seen in Table 3, which also reports the correlations between the dimensions of self-reported musical sophistication and the three scores (tonal, rhythm, total) from the AMMA listening test.

**Table 3:** Test-retest correlation for subscales of the Gold-MSI self-report inventory.

	Test-retest correlations (n = 53)	AMMA tonal score(n = 44)	AMMA rhythm score(n = 44)	AMMA total score(n = 44)
Active Engagement	.899**	.368*	.427**	.414**
Perceptual Abilities	.894**	.486**	.485**	.510**
Musical Training	.974**	.412*	.420**	.433**
Singing Abilities	.940**	.393**	.438**	.430**
Emotions	.857**	.305*	.323*	.332**
General Musical Sophistication	.972**	.463**	.502**	.503**

Footnote. Values of Pearson’s correlation coefficient are reported for test-retest reliability and correlations with the Advanced Measures of Musical Audiation (AMMA). *indicates a p-level of <.05 and ** a level of <.01.

The correlations between the self-report inventory and the test scores of the AMMA were all in the range of.30 to.51, which is in the upper range of what is usually reported as the correlation between a ‘paper-based’ self-report measure and actual perceptual or cognitive ability tests [87]. In particular the high correlations between the AMMA scores and self-estimated perceptual abilities as well as the general musical sophistication scores are very encouraging and even suggest that the new self-report inventory can potentially serve as a surrogate when perceptual testing of musical abilities is not available.

#### Study 3c

According to Werner, Swope, and Heide [22], the six subscales of the MEQ are grouped into two larger scale factors. Factor 1 is termed “Subjective/Physical Reactions” and includes subscales Affective Reaction, Positive Psychotropic effects, and Reactive Musical Behaviour. MEQ’s Factor 2 is termed Active Involvement and subsumes subscales Commitment to Music, Innovative Musical Aptitude, Positive Psychotropic Effects, and Social Uplift. On the face of the definitions of the subscales given by Werner et al. ([22] p.331), the MEQ’s Commitment to Music and Innovative Musical Aptitude scales seemed the most likely candidates to relate to the concept of musical sophistication in general, and to the Gold-MSI subscales Active Engagement and Musical Training in particular.

The correlations between the six MEQ subscales and the scales of the Gold-MSI are given in Table 4.

**Table 4:** Correlations between subscales from MEQ and Gold-MSI.

	ActiveEngagement	PerceptualAbilities	MusicalTraining	SingingAbilities	Emotions	GeneralSophistication
Commitment to Music	.241**	.206*	.223*	.292**	.255**	.309**
Innovative Musical Aptitude	.203*	.319**	.395**	.422**	.189*	.449**
Social Uplift	.111	.168	.139	.289**	.159	.229*
Positive Psychotropic Effects	.181*	.200*	.198*	.300**	.237**	.282**
Affective Reactions	.076	.146	.142	.222*	.142	.182*
Reactive Musical Behaviour	.126	.195*	.198*	.312**	.159	.264**

Footnote. Values of Pearson’s correlation coefficient are reported for correlations between the six dimensions (rows) of the Music Experience Questionnaire (MEQ) and the 5+1 dimensions of the Gold-MSI. * indicates a p-level of <.05 and ** a level of <.01.

All correlations between the subscales of the musical self-report inventories were of a low to moderate magnitude, which indicates that the inventories measure somewhat related, but certainly not identical constructs. Among all MEQ subscales, the Innovative Musical Aptitude scale, which includes ‘self-reports of musical performance ability’, is the one that correlated most highly with Gold-MSI subscales. This is not surprising since this is the only subscale that assesses self-reported abilities and skills at different levels. As expected, it correlated with General Musical Sophistication, and Musical Training as well as Singing Abilities, but only at a moderate level of about.4. While the MEQ’s Commitment to Music showed significant correlations with all Gold-MSI subscales, it had only a very moderate, albeit significant correlation, with the Gold-MSI’s Active Engagement scale (*r* = .241). The correlation between the Gold-MSI Emotions subscale and the MEQ’s Affective Reactions reached only.142 and was not significant, suggesting that the skills of emotional usage of music measured by the Gold-MSI are only weakly related to the more passive Affective Reactions measured by the MEQ.

Overall the results from Study 3c suggest convergent validity with the MEQ subscale *Innovative Musical Aptitude*, and discriminant validity with regards to constructs that clearly have little in common with the concept of musical sophistication, as indicated for example by the low correlations with the MEQ subscales Social Uplift, Affective Reactions, and Reactive Musical Behaviour, despite the fact that both inventories operate in the same domain.

#### Study 3d

The pattern of correlations between the five Gold-MSI subscales/general factor and the ‘Big Five’ from the TIPI is given in Table 5.

**Table 5:** Correlations between ‘Big Five’ personality traits as measured by the TIPI and Eysenck’s Extraversion scale and the subscales of the Gold-MSI self-report inventory.

	M (SD)	ActiveEngagement	PerceptualAbilities	MusicalTraining	SingingAbilities	Emotions	GeneralSophistication
Big Five							
Extraversion	9.2 (2.9)	.204**	.281**	.266**	.343**	.181*	.325**
Agreeableness	10.0 (2.3)	.103	.187**	.102	.188*	.136*	.177*
Conscientiousness	10.0 (2.9)	−.128	−.076	−.117	−.123	−.161*	−.164*
Emotional Stability	9.0 (2.8)	.083	.180*	.131	.132	.035	.159*
Openness	10.6 (2.3)	.392**	.361**	.296**	.326**	.409**	.428**
Eysenck							
Extraversion	8.2 (2.3)	.325**	.307**	.186*	.438**	.282**	.345**

Footnote. For all correlations n = 224, except for those involving Singing Abilities, where n = 161 due to a technical error. Means and standard deviations of the summed personality scores (range TIPI: 2–14, range Eysenck: 0–12) are also given. * indicates a p-level of <.05 and ** a level of <.01.

Table 5 demonstrates an interesting pattern of relationships between personality traits and aspects of musical abilities. Openness and Extraversion seem to be at least moderately related to all facets of musical sophistication and almost all correlations were highly significant. The extraversion scale from the TIPI and from Eysenck’s scale yielded very similar results, both correlating positively with all aspects of musical sophistication. Thus, for our sample of non-specialists we cannot find any support for the notion that higher levels of introversion help the development of musical abilities [118]. Interestingly, conscientiousness showed a negative, albeit very low, correlation with all facets of musical sophistication that does not support the wide-spread belief that skilful engagement with music requires a high degree of conscientiousness. Agreeableness and Emotional Stability were positively related to musical sophistication but correlations were generally lower than for Extraversion and Openness. The high correlation between Openness and musical sophistication is in line with the fact that Openness is commonly found to be the strongest correlate with achievements in tests of cognitive ability [87], and is also very much in line with the literature discussed above that highlights the close links between musical engagement and Openness, almost to a degree where musical behaviour becomes a constituent of this personality trait.

Taken together, the data of Study 3d suggest that individuals who are open to new experiences and rank highly on extraversion possess high levels of musical sophistication. It is however unclear whether openness and extraversion are a cause or effect of more frequent and more intense musical behaviours, along with the resulting higher levels of musical sophistication.

## Study 4: Self-Reported Musical Sophistication and Objective Listening Tests in a Large Sample

The Gold-MSI self-report inventory measures self-assessed levels of musical abilities, skills, and the degree of sophistication in musical behaviours. In Studies 1 to 3 we showed that the Gold-MSI has good psychometric properties with regards to the content validity of its individual items, the construct validity of its subscales as tested on a very large sample, the test-retest reliability, and with regards to concurrent and discriminant validity as evidenced by the correlations with other related and less related scales and musicality tests. In addition, we have been able to gain some insight into the relationships that musical sophistication has with other psychological constructs such as personality traits and general musical behaviours.

Having obtained a valid and reliable self-report measure of sophisticated musical behaviour, our next goal was to investigate which musical production and perception skills benefit from, or are at least associated with, which musical behaviours. Hence Study 4 compared scores from the Gold-MSI self-report inventory with the results of two specific listening tests across a large sample to assess the correlation between musical abilities and self-reported levels of musical sophistication. The two listening tests were chosen to assess distinct musical abilities that can be thought of as being very different but similarly important musical skills, namely melodic memory and musical beat perception. The nature of this set of tests is different from educational musical aptitude tests, such as the AMMA used above, which aim to test the general aptitude of students for musical achievement (in traditional Western music education).

In contrast with the AMMA, the two tests reported here relied mostly on excerpts from naturalistic musical stimuli. Both tasks test the ability to focus on a certain musical parameter (i.e. pitch interval structure, the musical beat) in the context of many other concurrent musical parameters. Neither of the response procedures required specialist music knowledge, which made the tests suitable for the general adult population. Both tests were modelled on well-known test procedures from the music cognition literature where the underlying cognitive mechanisms and experimental factors that affect test scores are well-understood (see descriptions and references to prior studies for each test in the Method section below).

### Method

#### Participants

We used the combined training- and test datasets which comprised all 148,119 participants (with useable data) who had taken the BBC’s *How Musical Are You?* online test in 2011. Whereas almost all participants had completed the self-report inventory (n = 148,037), we had slightly fewer participants for the two listening tests, namely 139,481 for the beat perception test, and 138,469 for the melodic memory test. 134,984 participants provided complete data for all two tests plus the self-report inventory. In fact, the participants of the *How Musical Are You?* online test also took a sound similarity as well as a beat production test, the results of which are not reported in this current paper.

The demographic statistics of the subset of participants used in Study 4 are virtually identical to the figures given in Study 1. In addition, a study was carried out to assess test-retest reliability and concurrent validity with the relevant subscales from the self-report inventory under more controlled conditions. 48 (test session) and 39 (retest session) participants were tested through an online interface at their homes. 34 individuals (16 women) with a mean age of 36.9 year (*SD* = 15.1) completed both test sessions which were 23 days apart on average (*SD* = 9.2, range: 10 to 64 days).

#### Melodic memory test: materials and procedure

Memory for melodies and tone sequences has been tested extensively for more than 50 years (see [119] for an early paper, and see [120] for a recent summary). In addition, most established musical aptitude tests include a melody memory subtest as a core component [7], [9]–[11], [121]. A very common paradigm is based on a same-different comparison of two short melodies, where participants have to judge whether the two melodies played successively are identical or different (in one or more notes). Thanks to the large number of publications using this paradigm, the cognitive mechanisms and determinants of melodic memory are fairly well understood [122]. The test battery is inspired by the cognitive paradigms used by Cuddy and Lyons [123] as well as Dowling and Bartlett [122]. Based on their findings, we designed a set of stimuli that balance several factors that have been shown to influence melodic memory, i.e. preservation of the contour of the intervallic structure vs. violations of contour, in-key vs. out-of-key errors, and near key vs. far key transposition distance (along the circle of fifths). The test battery uses the same AB comparison paradigm that has been used in previous cognitive studies [122]: each item consisted of two short melodies (containing between 10 and 17 notes) with the second melody always transposed and presented at a different pitch level than the first one. Harmonic distance, as measured on the cycle of fifths, was balanced across trials by presenting the second melody transposed either by a fifth or by a semitone. Participants were required to indicate whether the two tunes had an identical pitch interval structure or not, and to rate the confidence of their judgement on a 3-point scale (“I’m totally sure”, “I think so”, “I’m guessing”). Confidence ratings were not used for the derivation of the participants’ accuracy or d’ scores. 12 melody items were newly created following the approach described by Halpern, Bartlett, and Dowling [124] for generating novel melodic stimuli on the basis of the distributions for pitch intervals and tone durations from existing and well-known Western folksongs. The 12 trials consisted of 6 different- and 6 same-tune trials. The manipulations of the 6 different-tune trials comprised three melody items where melodic contour (and interval) was changed and three items where contour was preserved and only the pitch interval structure was changed. All manipulated items had two notes changed and overall item difficulty was calibrated in a small pilot sample. Participants were presented with two training items at the beginning of the test where the concept of transposition was explained in lay terms and the correct answer was given for each item. Items were screened individually for their contribution to the reliability of the overall test which led to the exclusion of one item that contributed negatively to the tests’ reliability as measured by Cronbach’s alpha. The alpha coefficients from the test and the retest sessions were.61 and.68 for the resulting 11-item testset. Test-retest reliability was computed from the participants’ *d’* test scores using Pearson’s r and Spearman’s rho correlation coefficients as well as the single-measure intra-class correlation coefficient (ICC) with a 2-way random model with absolute agreement (ICC = .54, *r* = .57, rho = .60, all *p*<.001). The psychometric properties of the melodic memory and beat perception tests have subsequently been optimised since the *How Musical Are You?* data were collected. As a result of several lab studies we have been able to create versions of the tests that are shorter in length and have better psychometric properties. The details of the test optimisations and results from the lab studies are currently being written-up in separate manuscripts. Therefore, for use in future research we recommend using the optimised versions of the tests, which have been compiled as version 1.0 of the Gold-MSI test battery and are fully documented and freely available from http://www.gold.ac.uk/music-mind-brain/gold-msi/. For more details on individual stimulus generation and all stimuli in music notation see Müllensiefen et al. [65].

#### Beat perception test: materials and procedure

Beat perception was assessed via a newly created variant of the Beat Alignment Test [125]. The test required participants to listen to 18 short instrumental excerpts (10–16 seconds). Tracks were overlaid with a metronome-like beep track that was exactly on the implied beat of the music for half of the items, or manipulated in one of three ways for the other half of the items: phase shift by 10% or 17.5% of the beat period, or tempo alteration by 2% relative to the beat of the music track. The participants’ task was to indicate whether the beep track coincided with the beat of the music or not, and to rate their confidence on the same scale used for the melody memory task (again, confidence ratings were not used for the derivation of the participants’ accuracy or d’ scores). The 18 tracks were taken from 9 different musical pieces belonging to three different genres (rock, jazz, and popular classical). The tempo of the musical pieces varied between 85 and 165 beats per minute. Six of the musical pieces were in duple meter while three items (one from each genre) were in triple meter. Items were screened individually for their contribution to the reliability of the overall test which led to the exclusion of three items that contributed negatively to the tests’ reliability as measured by Cronbach’s alpha. For the 15-item testset, the alpha coefficients from the test and the retest sessions were.87 and.92. Test-retest reliability was computed from the *d’* scores (ICC = .63, *r* = 70, rho = 72, all *p*<.001). Again, for use in future research we recommend using the optimised versions of the beat perception test which is part of version 1.0 of the Gold-MSI test battery and fully documented and freely available from http://www.gold.ac.uk/music-mind-brain/gold-msi/. For links to the soundfiles of the nine original music pieces see Müllensiefen et al. [65].

### Results and Discussion

For both tests, the overall mean accuracy scores were in a middle range between chance level (50% accuracy) and a perfect score. For the melodic memory test mean accuracy was.75 (*SD* = .17) and *d’*, a bias-free measure of performance, was at 1.55 (*SD* = 1.10). Mean accuracy for the beat perception task was.77 (*SD* = .16) and *d’* was 1.70 (*SD* = 1.19). Accuracy and *d’* scores for were highly correlated (*r* >.98) for both tasks. We therefore mainly report the conceptually simpler accuracy scores in the following results.

The correlation between the performances on both tests was very moderate (*r* = .26 for the accuracy scores and *r* = .27 for the *d’* scores), indicating that the two tests largely measure different abilities. The correlation between the performances on both tests was very moderate (*r* = .26 for the accuracy scores and *r* = .27 for the *d’* scores), indicating that the two tests largely measure different abilities. This low correlation between the two tests does not suggest the creation of a combined sum-score for measuring general musical sophistication from perceptual tests. In addition, we believe that musical sophistication is a broader psychological attribute that comprises more than melodic memory and beat perception ability and, while we are ultimately interested in a single perceptual index, we will explore carefully in a future study with a more comprehensive perceptual test battery whether the perceptual data can be modeled with the help of a general musical sophistication latent factor.

Table 6 shows the correlation between the scales of the self-report inventory and the scores on the listening tests. The table contains the correlations from the large online sample as well as from the smaller sample of the test-retest study.

**Table 6:** Correlations between sub-scales of the self-report inventory and performance on the two listening tests.

	Active Engagement	Perceptual Abilities	Musical Training	Singing Abilities	Emotions	General Sophistication
Listening Tests						
Online Sample						
Melodic Memory	.103***	.261***	.301***	.259***	.128***	.285***
Beat Perception	.224***	.342***	.356***	.305***	.218***	.375***
Test-Retest Sample						
Melodic Memory	.344*	.407*	.521**	.358*	.423**	.511**
Beat Perception	.216	.325	.354*	.353*	.308	.379*

Footnote. Sample sizes differed slightly between bivariate correlations from the online sample and ranged from n = 136,924 to n = 139,062. Sample size for the test-retest sample was n = 34.

As expected, the highest correlations were obtained with the musical training and perceptual abilities subscales, as well as with the general musical sophistication scale. In particular, the correlations of self-reported general musical sophistication with beat perception (*r* = .38) and melodic memory performance (*r* = .51) obtained from the test-retest sample indicate a convergent validity of self-report inventory and perceptual tests. The magnitude of these correlations is in a similar range to the correlations between self-report inventory and the scores on the AMMA musicality test in Study 3b (*r* = .50 for General Musical Sophistication and AMMA rhythm score and *r* = .46 for General Musical Sophistication and AMMA tonal score, see Table 3). This suggests that the lower correlations obtained from the large online sample are at least partly due to the difference in testing conditions. We had no control over the conditions under which the large sample of participants of the *How Musical Are You?* study took the listening tests. A decrease in effect size between controlled lab experiments and uncontrolled online studies is fairly common and has been reported repeatedly [126]–[129]. However, in practice the greater amount of noise in online data is often compensated for by larger sample sizes. Indeed, the sample size of the *How Musical Are You?* study is several orders of magnitude larger than both the sample from Study 3b, and also the test-retest study validating the two listening tests.

**Figure 2:**
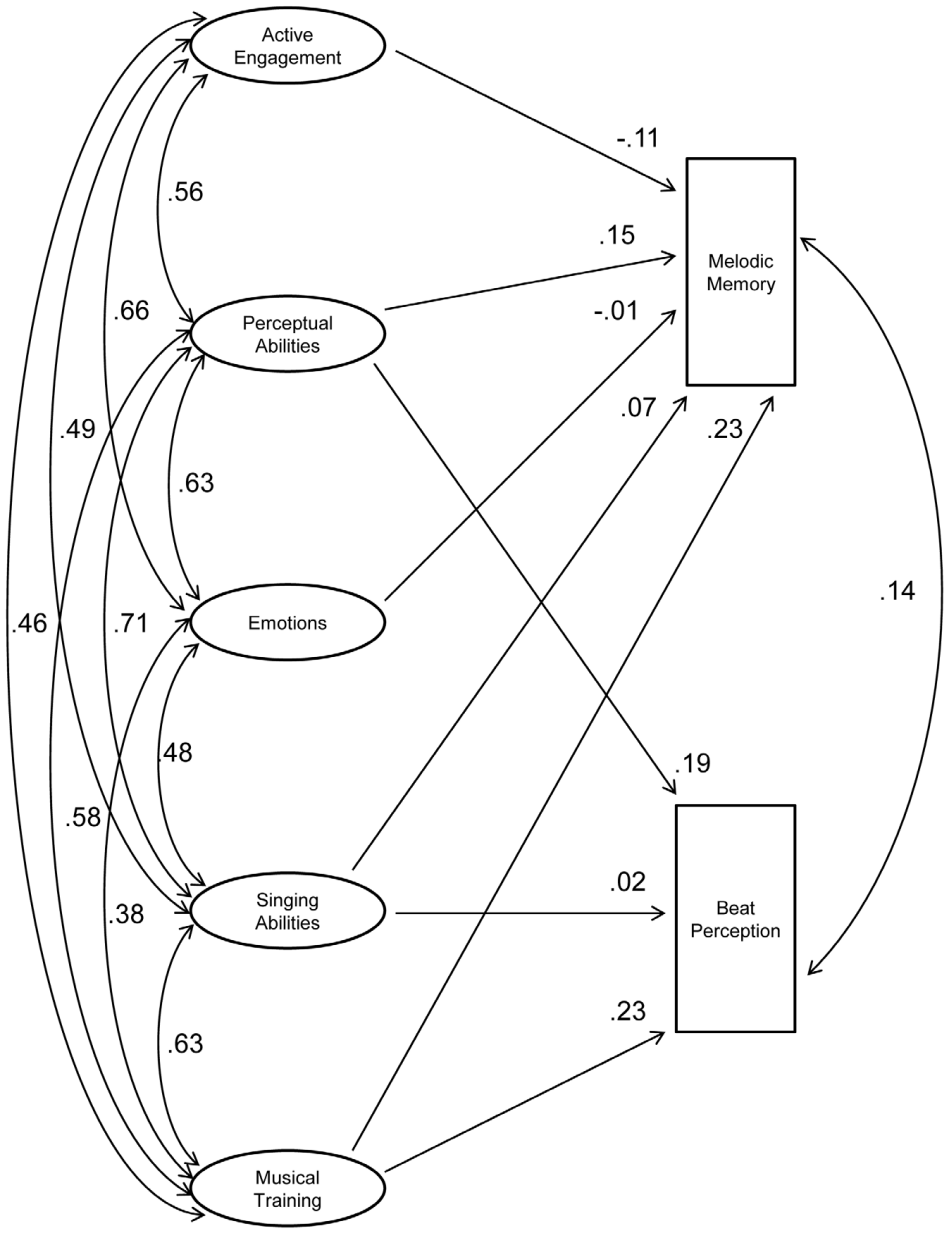
Structural equation model relating subscales of the self-report inventory to performance scores on the two listening tests.

We derived a structural equation model from the correlations between the five dimensions of musical sophistication and the scores on the two listening tests. The model included all inter-subscale and inter-test correlations as well as paths from all 5 subscales to each listening test. We removed two paths with non-significant parameter estimates and the resulting model fitted the data extremely well (χ^2^ = 1.92, df = 2, *p* = .384; TLI = 1, CFI = 1, RMSEA <.001, SPMR <.001), and is graphically depicted in Figure 2.

As the regression weights in Figure 2 show, Musical Training and Perceptual Abilities have relatively strong relations with the beat perception and melody memory tests. Performance on both tests clearly benefits from the amount of musical training an individual has had. Self-reported perceptual abilities also have a significantly positive relation with performance on the two listening tests and, as expected, singing abilities are also positively related to melodic memory performance, but only to a small degree to beat perception. Interestingly, the influence of active engagement and emotional musical sophistication on melodic memory scores is negative once the influence of all other dimensions of musical sophistication is controlled for. This suggests that detecting fine differences between different versions of the same melody is a skill that depends to a large degree on instrumental training and conversely, that high levels of listening engagement and a focus on the emotional functions of music might not be helpful when the task is to focus on subtle differences in melodic structure.

**Figure 3:**
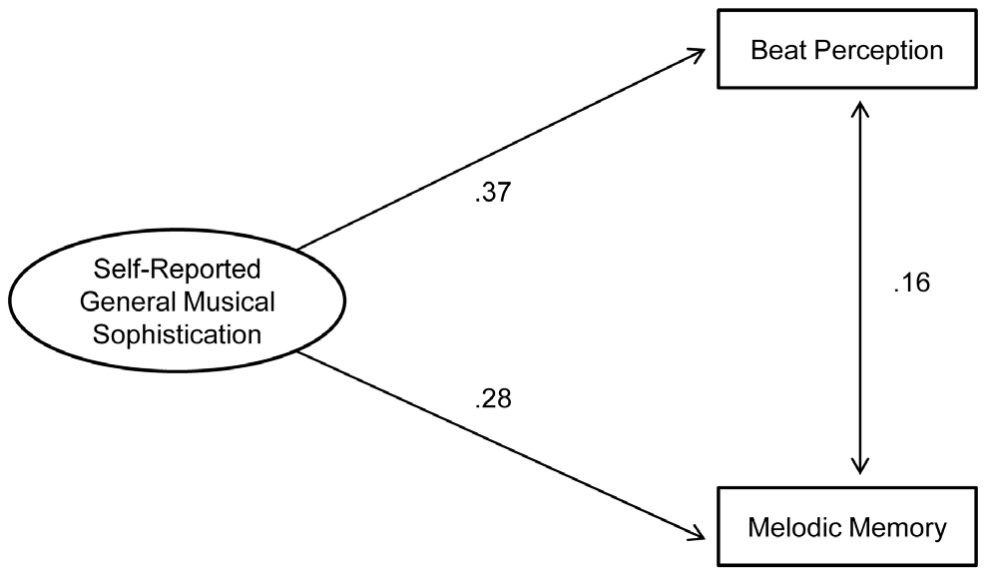
Structural equation model demonstrating the influence of self-reported general musical sophistication on the performance on the objective listening tasks.

We constructed a second structural equation model relating General Musical Sophistication to the performance on the two listening tests. The model fitted the data very well (indices were indicating essentially a perfect fit) and is graphically shown in Figure 3. General Musical Sophistication was positively related to both listening tasks and relatively strong regression coefficients were obtained for beat perception (.37) and melodic memory (.28), while the correlation between both tests after accounting for self-reported General Musical Sophistication was fairly low (.16).

In summary, the results of Study 4 show that the general musical sophistication scale indexes both listening tasks positively, and we can hence speak of a coherent set of tests. This is despite the fact that the two tasks measure very different musical skills.

## Study 5: The Socio-Demographic Conditions of Musical Sophistication

Studies 1 to 3 established a new self-report inventory of musical sophistication while Study 4 compared self-reported musical skills and behaviours to objective tests of two different musical abilities. The aim of Study 5 was to determine the degree to which musical sophistication and performance on the listening tests are associated with socio-demographic variables once the degrees of musical training and of active engagement with music have been controlled for. We used the large sample gathered from the BBC’s *How Musical Are You?* online implementation. This implementation did not include a formal inventory of socio-economic status (SES), but it comprised several questions covering the social context of participants as well as aspects of education and occupation as core constituents of SES. With regards to wealth as the third chief constituent of SES, we were able to aggregate participants’ scores from the *How Musical Are You?* test at the level of British local authorities and compare them to income data available from the UK Office for National Statistics. In general, it is unclear whether there is a causal relationship between musical sophistication and socio-economic variables and what the directions of causes and effects are [130]. Thus, in the following analysis we alternate between a strictly correlational description and an analytical perspective in which musical sophistication represents the dependent variable and socio-economic factors act as independent predictors.

### Method

#### Participants

In order to work with a culturally homogeneous sample, we selected for this study only those 90,474 participants from the large Internet sample who had indicated that they were currently residing in the UK, and had spent the formative years of their childhood and youth in the UK. The mean age of the selected participants was 37.2 years (*SD* = 15.2) and 43.6% were female. For 70,097 of the British participants we had a valid postcode (with the last two digits truncated to preserve anonymity), and this allowed us to average participants’ test scores at the level of 379 local authorities in England, Scotland and Wales.

#### Materials and methods

Apart from age and gender, participants indicated the highest level of education obtained (6 categories) and/or the highest level of education they were expecting to achieve (5 categories), their ethnic group (9 categories), their occupational status (8 categories) and their occupation (24 categories). In addition to these socio-economic variables, we also included the subscale scores for musical training and active engagement as predictor variables.

These predictor variables were related to scores on the General Musical Sophistication factor and to the scores from the two listening tests described in Study 4. We split the sample of participants into a training- (n = 45,647) and a test (n = 45,482) dataset, and analysed the association of musical sophistication with socio-economic variables in three analysis steps.

First, we ran a random forest regression (using the R package *randomForest* for the computations [131]) on the training dataset to determine the relative importance of each socio-economic variable in predicting musical sophistication (see [132], for the initial concept of random forest classification and regression, and [133] for a summary overview). Random forests are able to make use of information in ‘weaker’ explanatory variables, in that they model complex variable interactions. They also have the additional advantage that results can be generalised to new datasets, because they do not tend to overfit on training data [134]. As a second analysis step we used conditional inference significance tests, implemented in the R package *coin* [135] and based on permutation statistics [136], as post-hoc tests to identify the categories within these variables for which significant main effects could be observed. Permutation tests do not make any distributional assumptions, but take the shape of the empirical distribution into account and are therefore not affected by large sample sizes or skewed distributions. We adjusted *p*-values for multiple comparisons using the ‘single-step’ procedure suggested by Westfall and Young [137]. In the third analysis step, the test dataset served to confirm the results derived from the training dataset and to summarise them in easily interpretable tree models based on recursive partitioning [138].

In sum the three analysis steps deliver different insights into this large and complex dataset: the random forest model indicates the importance of each variable, taking into account main effects as well as all complex variable interactions, whereas the permutation tests inform about the positive or negative main effects of each variable, and the tree model synthesises both approaches by picking the most important variables and partitioning the data into homogeneous subsets. Thus, the latter approach models interactions and indicates which combination of variables (or categories of variables) leads to higher versus lower musical sophistication and performance scores.

Finally, for self-reported musical sophistication scores as well as listening test scores we used the accompanying truncated postcodes of individuals to aggregate scores at the level of the 379 British local authorities via the geographical data of the Ordnance Survey [139]. This allowed us to correlate musical scores with the median weekly gross income as published in the Annual Survey of Hours and Earnings collected in 2011 by the Office for National Statistics [140].

### Results

Within the random forest model, the importance of each independent variable is computed as the percentage increase of the mean squared error in the dependent variable when the given predictor is excluded from the model. Table 7 reports the importance of the 8 socio-demographic variables and the two subscale scores for predicting self-reported General Musical Sophistication and the performance on the two listening tests. It is worth noting that, despite being recognised as a powerful statistical prediction model, the random forests including all socio-economic variables were only able to explain small proportions (i.e. between 4.7% and 13.6%) of the variance in the scores for self-reported musical sophistication and the two tests.

**Table 7:** Variable importance according to random forest model.

	General Musical Sophistication	Melody Memory	Beat Perception
Age	247	96	96
Gender	100	26	62
Ethnic Group	38	12	7
Occupation	263	76	73
Occupational Status	218	62	93
Level of Education Obtained	156	62	65
Level of Education Expected to Obtain	105	55	66
Musical Training	–	187	208
Active Engagement	–	32	48
*R* ^2^	.047	.110	.136

*Footnote.* Numerical values represent % increase in mean squared error if variable is omitted from model and hence higher values mean greater importance. Note that the model predicting general musical sophistication did not use the subscale scores for music training and active engagement.

#### General musical sophistication

Table 7 shows that the socio-economic variables most predictive of self-reported Musical Sophistication are Occupation, Age, Occupational Status, and Level of Education Obtained. According to the subsequent permutation tests, younger participants, participants working as music or media professionals or working in education, and participants currently at school or university, or having completed A-levels reported significantly higher levels of musical sophistication (values of the standardised test statistic and corresponding *p*-values from the permutation tests for the levels of all variables are given in Table S4 in File S1). In contrast, retired participants reported significantly lower levels of musical sophistication.

**Figure 4:**
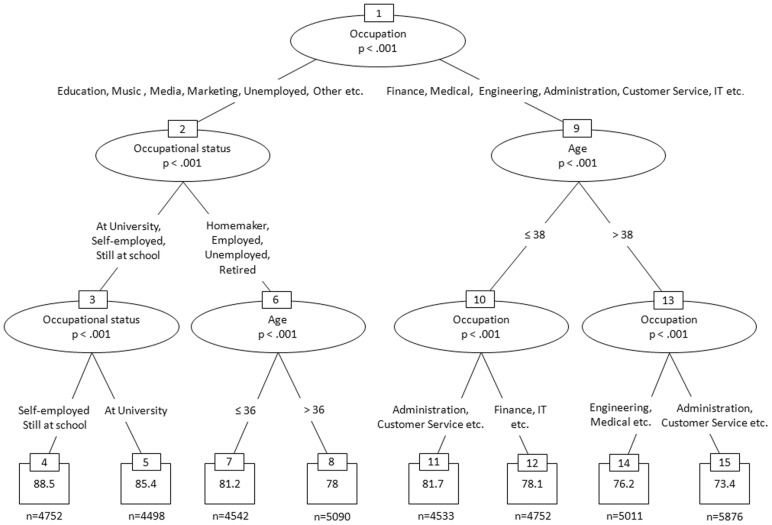
Conditional inference regression tree modelling general musical sophistication with variables of socio-economic status. The tree model is interpreted by starting at the top of the tree, following each branch down from each node, to arrive at a terminal node with the average scores given inside the squares on the graph. For example, descending to the right from node 1 (‘Occupation’) down the ‘Finance, Medical, Engineering, Administration, etc.’ branch, then descending to the right at node 9 (‘Age’) down the ‘>38’ branch, and finally descending the right branch (‘Administration, Customer Service, etc’) going off node 13 (‘Occupation’) to arrive at terminal node 15, this can be interpreted as follows: People working in administrative or customer service occupation and being older than 38 years will obtain on average a general musical sophistication score of 73.4. Technically, the logical combinations of these two conditions can be regarded as an interaction of the two predictor variables. The significance values for each split are given within the oval nodes and are derived from a Monte Carlo resampling procedure that adjusts for multiple testing.

These relationships were confirmed by the regression tree model run on the test dataset and are summarised graphically in Figure 4. We limited the depth of the tree to a level where terminal nodes would contain at least 10% of participants (after excluding participants with missing data from the sample). The graph shows that the highest level of self-reported musical sophistication (average score of 88.5) is found for participants who are either still at school or are working as self-employed, in education, media and music professions (node 4), while self-reported musical sophistication was lowest (average score of 73.4) for participants over the age of 38 working in administrative or customer service professions (node 15).

#### Melody memory task

The random forest analysis identified Musical Training, Age, Occupation, Occupational Status, and the Highest Educational Degree obtained as the five most important variables for predicting performance on the melodic memory task. Results from the permutation tests showed that older participants and participants who self-reported more musical training performed significantly better on this task. Several significant main effects for categories of occupational status, occupation, and education level obtained seemed to be related to this age effect, e.g. participants still at school or university or having only obtained school qualifications (GCSE, or A-level) scored significantly worse than expected. On the other hand participants with university degrees, those being in full-time employment or working as self-employed, and those working in education/training, media or music professions achieved significantly higher scores.

**Figure 5:**
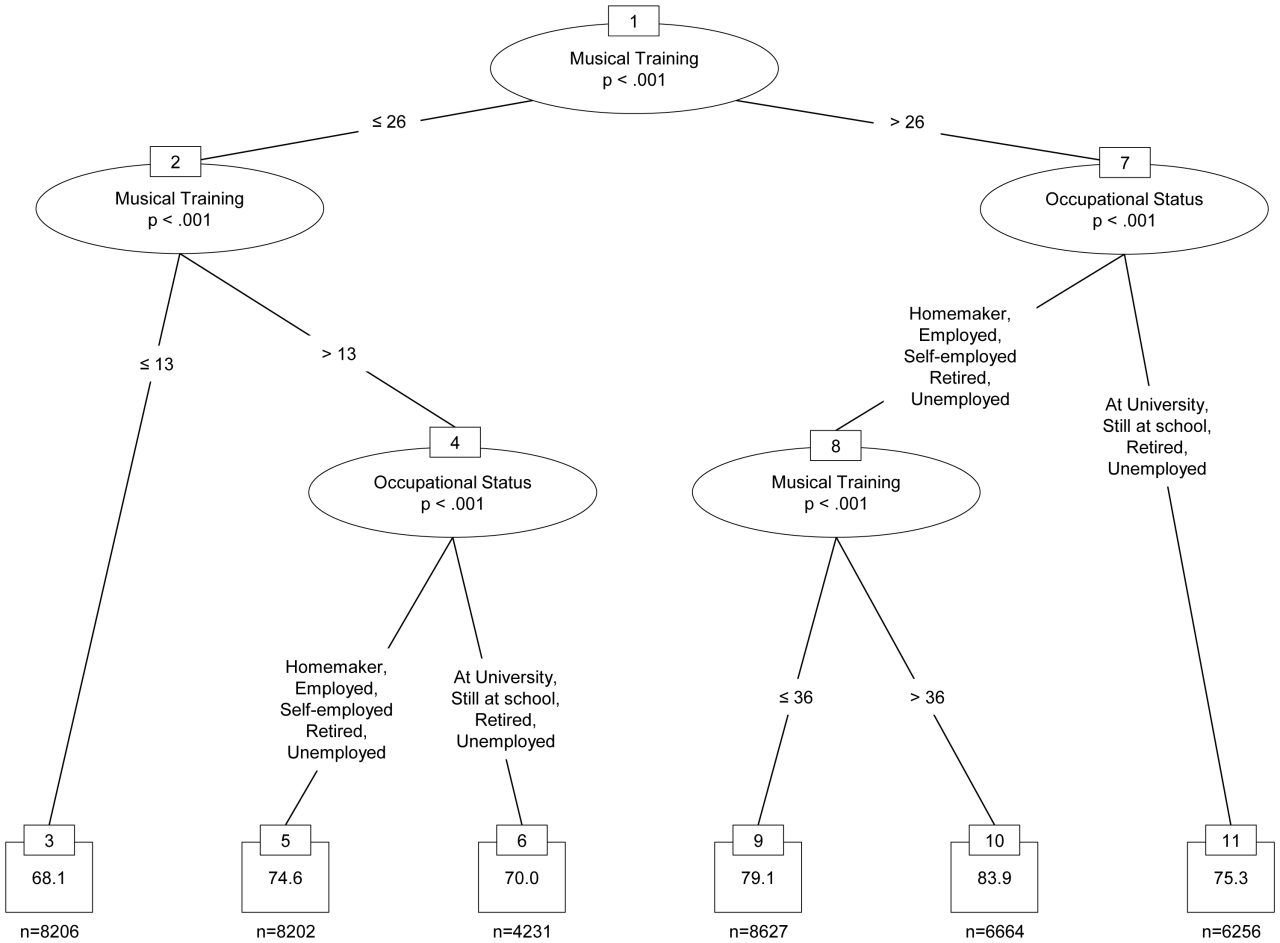
Conditional inference regression tree modelling accuracy scores (percentage scale from 0 to 100 where 50 indicates chance level) in the melody memory task using self-reported musical training and variables of socio-economic status as predictors.

The importance of musical training and certain categories of occupational status that are associated with older ages (e.g. employed, homemaker) for scoring high on the melodic memory task is reflected in the summarising tree model in Figure 5.

#### Beat perception task

**Figure 6:**
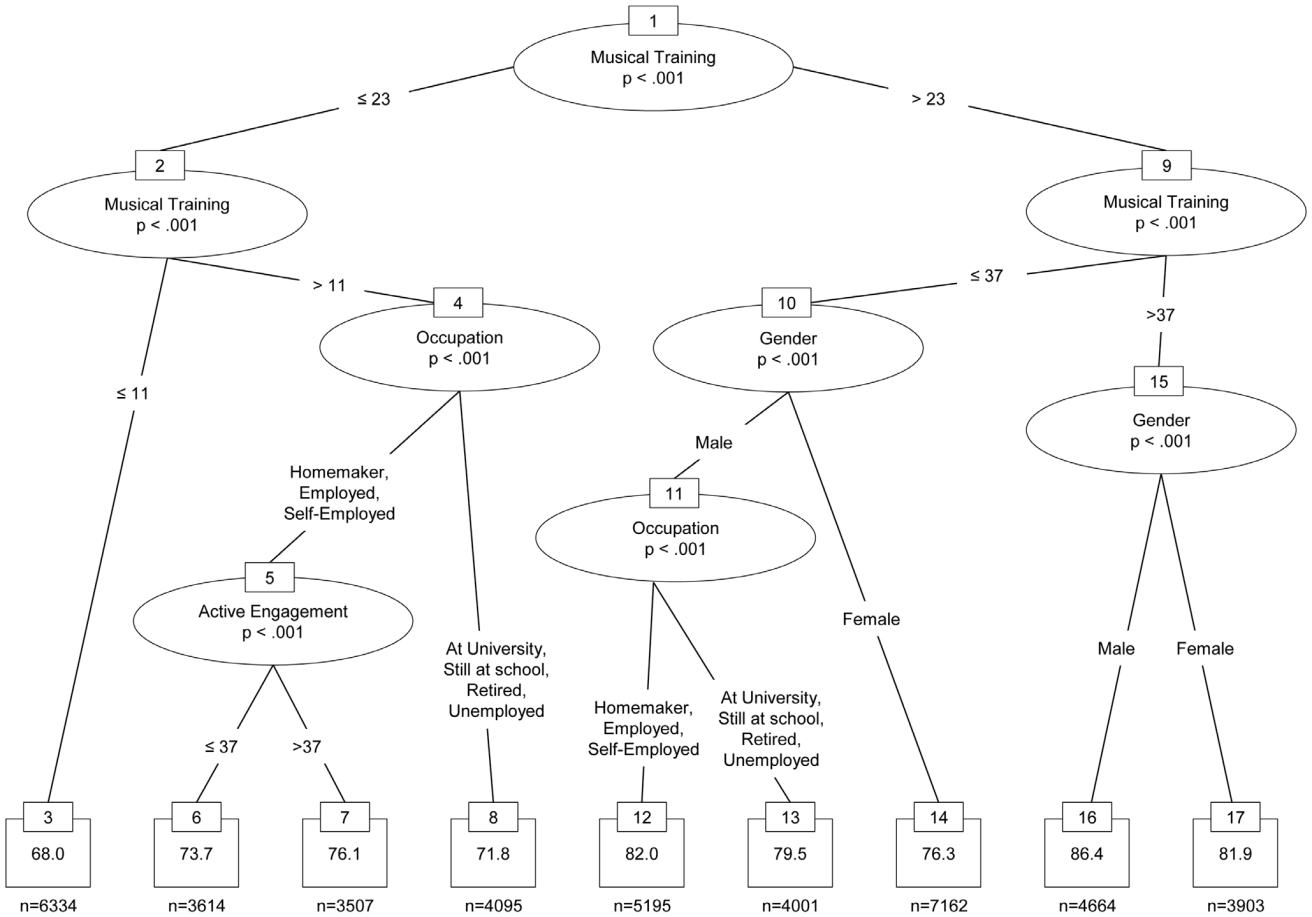
Conditional inference regression tree modelling accuracy scores (percentage scale from 0 to 100 where 50 indicates chance level) in the beat perception task using self-reported musical training, active engagement, and variables of socio-economic status as predictors.

According to the random forest analysis, the five most important variables for predicting performance on the beat perception task were self-reported musical training, age, occupational status, occupation, and the levels of education obtained and aspired to. The permutation tests indicated that musical training had a positive main effect on test scores but age was negatively related to performance on this task. Participants at university, in full-time employment, or those that were self-employed, especially those working in IT, media, or music professions scored better on this task while homemakers, retired participants, and those still at school or having obtained only a GCSE qualification scored significantly worse. Additionally, women achieved significantly lower beat perception scores than men. The tree model in Figure 6 summarises these findings and shows how other variables interacted with musical training, which was the most important variable for predicting beat perception abilities. For example, the graph depicts how, for low levels of musical training, more active musical engagement leads to better test performance (terminal nodes 6 and 7), and how musical training was beneficial for test performance for both genders despite an overall higher achievement level for men (terminal nodes 16 vs. 17).

#### Relating regional income to musical sophistication

Looking at the data across the 379 local authorities in the UK, we found several significant correlations with data from the national income survey. Table 8 shows that the highest correlations with median weekly gross income are for musical training, general musical sophistication, and the performance on the two listening tests. The amount of variance that regional income can explain in certain musical variables was fairly high, in particular with respect to the performance on the two listening tests where 8.3% (melodic memory) and 12.6% (beat perception) of the variance was accounted for by regional income as the only predictor variable. On the other hand, Active Engagement and Musical Emotions yielded near-zero correlations with median weekly income of the local authority.

**Table 8:** Pearson correlations across 379 local authorities between median weekly gross income and the subscales of the self-report inventory as well as the performance scores from the listening tests.

	Correlations w/weekly gross income (n = 379)	Adjusted R^2^
Active Engagement	.049	<.001
Perceptual Abilities	.173**	.027
Musical Training	.339**	.113
Singing Abilities	.150**	.020
Emotions	.024	<.001
General Musical Sophistication	.165**	.025
Melody Memory	.291**	.083
Beat Perception	.358**	.126

*Footnote*. Pearson’s correlation coefficients and adjusted R^2^ values from a linear regression model having only weekly income (in addition to an intercept) as predictor. *indicates a p-level of <.05 and ** a level of <.01.

**Figure 7:**
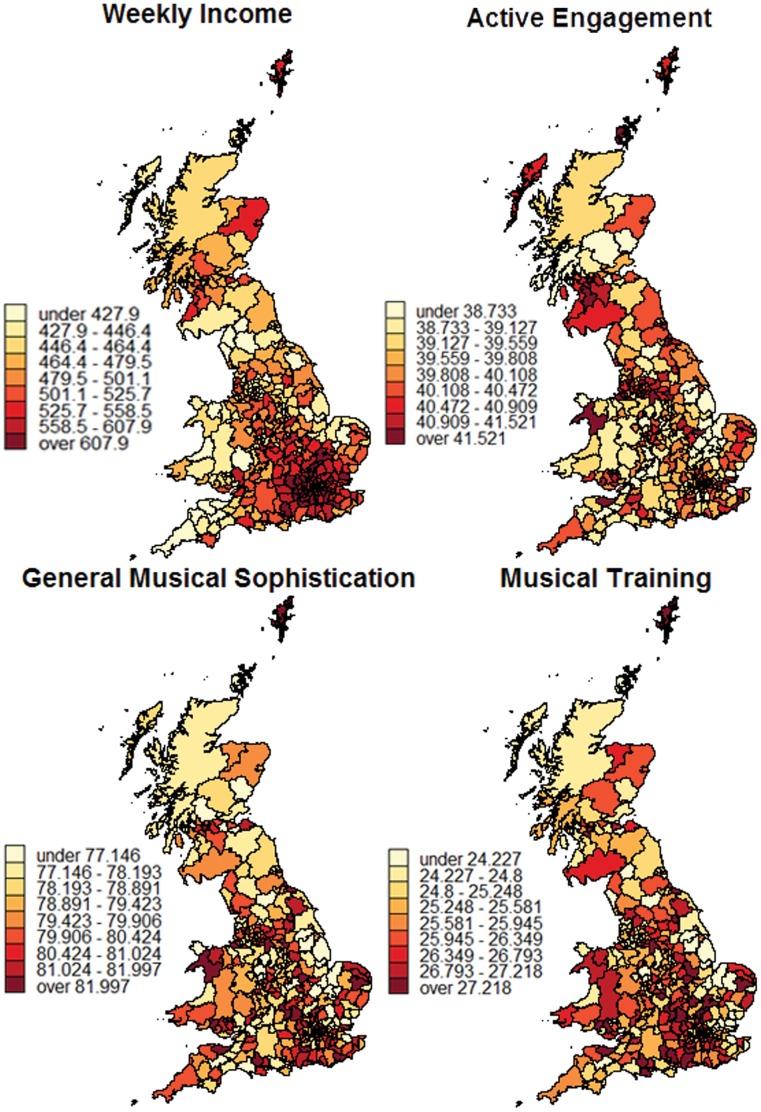
Distribution of median weekly gross income according to the 2011 Annual Survey of Hours and Earnings survey (Office for National Statistics, 2012) and general musical sophistication, musical training and active engagement across 379 local authorities of Great Britain. Values for all four variables were each split into 9 quantiles with approximately equal numbers of local authorities.

Because the correlations with income were obtained across geographical regions, it is possible to plot maps of the distributions of dimensions of musical sophistication and compare them to the distribution of regional income. Figure 7 shows that there is a clear concentration of high-income local authorities in and around London and the so-called ‘Home Counties’ (e.g. Buckinghamshire, Hertfordshire, Essex, Kent, Surrey, Sussex). The medium-sized correlations with musical sophistication and musical training are visible especially in urban areas in Scotland and Northwest England (Manchester and Liverpool). This seems to support the notion that certain types of musical engagement, especially musical training, are associated with greater wealth. However, the maps also show some clear differences between income levels and aspects of musical sophistication. For example, in the West Country and in parts of Wales, participants reported relatively high levels of general musical sophistication despite generally lower income levels. This might be due to regional musical traditions, such as choirs and amateur music ensembles, which are particularly strong in these regions ([141] p. 597).

Finally, the independence of active musical engagement (i.e. active musical listening, concert attendance, amount of money spent on music, reading and writing about music) from regional income (*r* = .049, n.s.) is clearly visible from the two respective maps. London and the Home Counties, the wealthiest regions in Great Britain, did not report particularly high levels of active musical engagement.

## Discussion

The first aim of this paper was to develop and evaluate a novel instrument for measuring self-reported individual differences in skilled musical behaviours in the general (i.e. non-specialist) population. We have termed this psychometric construct ‘musical sophistication’. Drawing on a very large data sample from a non-specialist adult population (n = 147,663), we found the construct to be best described as comprising five different factors in addition to one general factor that drives skilled musical behaviours on all dimensions. We implemented the five factors and the general factor as subscales and demonstrated that, with this 5+1 structure, the new self-report inventory possesses high internal consistency as well as test-retest reliability, and has been externally validated through comparisons with another music-related self-report inventory and a standard auditory musicality test. Having a reliable measurement instrument at hand then allowed us to investigate correlates and conditions of musical sophistication, in order to identify other aspects of human personality and behaviour that potentially interact with the development of musical skills.

In a separate but smaller sample (n = 224) we found significant correlations between Extraversion and all 5+1 subscales of the self-report inventory in line with previous research that reported a positive influence of high extraversion traits on different musical listening styles [113], [142]. These findings are in contrast with earlier claims [118] that high levels of introversion are more common in highly musically skilled individuals (the ‘bold introvert’ [106]). Given that these earlier studies exclusively recruited professional or semi-professional musicians, we suspect that introversion as well as higher levels of conscientiousness are only associated with high musical skills in the specialist population of (classical) professional musicians. However, our data indicates that for the non-specialist population skilled musical behaviour is positively correlated with extraversion and even more strongly with openness to experience.

The unique sample (n = 147,663) derived from the BBC’s online implementation of our test provided us with the opportunity to compare self-reported musical skills with the performance on two listening tasks: testing memory for melodies and the accuracy in the perception of a musical beat. A structural equation model showed that formal musical training has a positive influence on the ability to memorise melodies and on the perception of small deviations in musical timing. This is not surprising given that most methods of musical training in our cultural sphere focus on the accurate performance of musical structure (such as melody) and also emphasise the importance of an accurate musical pulse (e.g. for ensemble playing). In contrast, self-reported active musical engagement did not have a positive influence on the performance on the melodic memory test but it did affect the performance on the beat perception test positively, especially for those individuals with very low levels of musical training (on an instrument), as indicated by the regression tree model in Figure 6. Given that the active engagement subscale combines a number of activities related to focused music listening, we take this to suggest that active music listening and deliberate aural processing can train certain musical abilities even in the absence of formal musical training. This is in line with empirical evidence summarised in the introduction [57]–[59] showing that a range of musical skills are acquired through aural processing via statistical learning and leading to considerable amounts of implicit musical knowledge (see also Part 3 in [143]). Following this line of reasoning, an interesting avenue for future research would be to investigate whether it is possible to identify musical abilities that are enhanced by intensive listening behaviour but not by training on an instrument and vice versa.

Finally, we compared self-reported musical sophistication and performance on the two listening tests to socio-economic data from a sub-sample of British participants from the large-scale and online implementation. Overall, and despite the fact that we used a powerful data-mining technique, socio-economic variables were able to ‘explain’ only small proportions of the variance in the musical data. However, the variables with the strongest associations were related to occupation, occupational status, education, and age, while gender and ethnic group had far less predictive power. A possible interpretation of the influence of these variables on the self-report data is that musically sophisticated behaviour is strongly linked to an early stage in life when people are able to organise their time in a flexible way (e.g. when they are at school or university or when they are self-employed). This interpretation does not hold true for retired people, however, supporting the fact that age is an important factor, with younger ages reporting higher levels of musically sophisticated behaviour. In addition, certain professions that have a natural link with music (music, media, and educational professions) seem to extend the period of musically sophisticated behaviour beyond the early and flexible stage in life.

Music and media professions and self-employed or full-time working participants also generally achieved the highest scores across the listening tests. But performance on the tests was partly related to other socio-economic variables as well, and we found some differences between the two tests. Increased age was associated with a better performance on the melodic memory test, while younger participants did better on the beat perception test. These differences might be explained partly by a cohort effect of musical listening styles (beat-based vs. melody-focused) that may differ for the different age groups gathered in this sample [144].

We interpret these results from developmental perspective suggesting that musically sophisticated behaviour often develops at an early and flexible stage of life (end of secondary school to end of undergraduate university degree or beginning of working life) where most people have the time and motivation to engage with music in sophisticated ways, including musical training on an instrument and extensive listening engagement. Along with the musical training received in this phase, skills on an instrument are acquired and certain auditory skills such as melodic memory are trained by extension. At least some of the acquired skills are retained in older age and remain with the individual beyond the period of high musical engagement. This interpretation can explain the positive effect of age on the melodic memory task. In contrast, it is possible that other skills, such as the ability to detect subtle deviations from a musical beat, require continued sophisticated engagement with music to be preserved. A longitudinal study would be necessary to determine whether aural skills like beat perception are diminished as the effects of musical training and active engagement with music are gradually reduced across the life span, or whether the cohort effects of familiarity and listening styles are responsible for the differences in performance that we found in this cross-sectional study. Similarly, further work is needed to understand the interesting gender differences found in the beat perception task.

The clear and significant correlations between several facets of self-reported musical sophistication (i.e. musical training, perceptual abilities, general musical sophistication, singing abilities) and the performance on the two listening tasks on the one hand, and income at the regional level on the other hand are surprising and also merit further investigation in future studies. The direction of the influence between these variables is not clear from an a priori perspective. It is worth noting that the adult participants of the *How Musical Are You?* test were only asked to enter their current postcode. Therefore, it is impossible to evaluate from this individual correlation whether participants had received more musical training because they live in a more wealthy area or whether musical training did in any way support their professional development such that they achieved a higher socio-economic status and settled in more wealthy areas. A third, and perhaps more likely explanation, is that a common factor drives both wealth/socio-economic status on one hand, and also musical training/sophistication on the other. This common factor could be general cognitive ability or intelligence, which has been shown to correlate with musical training and academic achievements in a number of previous studies [130], [145]. However, considering the significant correlations between listening test scores and regional income, other possible common factors could include personality traits such as competitiveness, general test taking abilities or support from parents in early life stages, which might have had a positive influence on both active engagement with music and academic/professional achievements (see [146]–[147] for suggestions of similar explanatory mechanisms).

In conclusion, this paper makes three contributions to the field; firstly, we have developed ‘musical sophistication’ as a concept for describing the different types (facets) of skilled musical behaviour in the general population of Western societies. Secondly, we have used a large sample of participants to develop the Goldsmiths Musical Sophistication Index as a new self-report inventory that quantifies musical sophistication in its different facets. The Gold-MSI is a multidimensional construct that covers very different facets of skilled musical behaviour, but data analysis showed that there is also a general factor of musical sophistication that arises from the correlations between these various facets. The Gold-MSI has been calibrated to capture the large variations in musical skills and expertise found in the general population, including non-musicians. Moreover, Gold-MSI scores are related to performance on a number of objective listening tests. Thirdly, we have investigated psychological correlates and socio-demographic contexts of musical sophistication with the aim of elucidating the conditions that are associated with individual differences in musical sophistication in general. We found musical sophistication to be related to certain personality traits (foremost, openness to experience and extraversion) and also to be associated with socio-demographic and socio-economic markers. These markers point to a stage in late adolescence and early adulthood where sophisticated engagement with music peaks for large parts of the population. For older participants, we found the extent of musically sophisticated behaviours to be generally lower, unless individuals have the opportunity through their profession (e.g. educational, media, and music-related professions) to maintain engagement with music at a high level. We therefore believe that the concept of musical sophistication, as implemented in the Goldsmiths Musical Sophistication Index, is a robust and comprehensive empirical construct that is directly related to real-world experiences in Western societies.

Returning to the title of this paper–The musicality of non-musicians–we are able to conclude that musical sophistication varies across the general population of Western societies and people differ greatly in the types and extent of skilled musical behaviours that they report, as well as in the musical listening skills that we were able to measure. However, we found that musical listening skills and musical behaviours are very clearly related, and our data support theories of explicit as well as implicit learning of music, while demonstrating the extent to which sophisticated engagement with music is very much part of people’s social reality.

## Supporting Information

File S1 **Table S1, Items of self-report inventory.** Values of Cronbach’s alpha are derived from the full sample of 147,633 participants. **Table S2, Inter-factor correlations for confirmatory model 4. Table S3, Data norms for subscales and general sophistication (sample n = 147,633). Table S4, Values of the test statistic and corresponding p-values derived from the conditional inference permutation tests for all socio-economic variables as well as self-reported musical training and active engagement influencing General Musical Sophistication scores as well as performance on the two listening tests.**
(DOCX)

Textual Description S1 (DOC)
