# Correction: The Musicality of Non-Musicians: An Index for Assessing Musical Sophistication in the General Population

**DOI:** 10.1371/journal.pone.0101091

**Published:** 2014-06-24

**Authors:** The PLOS ONE Staff

In the third paragraph of the Results and Discussion section under Study 2: Assessing the Adequacy of the New Self-Report Inventory, the reference should be “(see Table 1).”

In the first paragraph of the Results and Discussion section of Study 3: Reliability, Validity and Correlates of the Self-Report Inventory, the second sentence should read: “As Table 2 shows, all scales possess good or very good estimates of internal reliability.”

In the first sentence of the fourth paragraph under the heading for Study 3: Reliability, Validity and Correlates of the Self-Report Inventory, the wrong name is used for the questionnaire. The correct name is the “Musical Experience Questionnaire.”

Table S4 in File S1 is missing a decimal separator and numerical values. Please see below for a complete File S1 with corrected Table 4.

## Supporting Information

File S1 **Table S1, Items of self-report inventory.** Values of Cronbach’s alpha are derived from the full sample of 147,633 participants. **Table S2, Inter-factor correlations for confirmatory model 4. Table S3, Data norms for subscales and general sophistication (sample n  = 147,633). Table S4, Values of the test statistic and corresponding p-values derived from the conditional inference permutation tests for all socio-economic variables as well as self-reported musical training and active engagement influencing General Musical Sophistication scores as well as performance on the two listening tests.**
(DOCX)
