# Supplementary material for: The Musicality of Non-Musicians: An Index for Assessing Musical Sophistication in the General Population
Source: PLoS One. 2014 Feb 26;9(2):e89642. doi: 10.1371/journal.pone.0089642 (PMC3935919; doi:10.1371/journal.pone.0089642)
Supplement: File S1 — Table S1, Items of self-report inventory. Values of Cronbach’s alpha are derived from the full sample of 147,633 participants. Table S2, Inter-factor correlations for confirmatory model 4. Table S3, Data norms for subscales and general sophistication (sample n = 147,633). Table S4, Values of the test statistic and corresponding p-values derived from the conditional inference permutation tests for all socio-economic variables as well as self-reported musical training and active engagement influencing General Musical Sophistication scores as well as performance on the two listening tests. (DOCX) [file pone.0089642.s001.docx]

Table S1

| Items of the self-report inventory |
| --- |
| **Active Engagement (9 items, α=.872)** |
| I don`t spend much of my disposable income on music |
| I enjoy writing about music for example on blogs and forums |
| I have attended ___ the following number of live music events as an audience member in the past twelve months |
| I keep track of new music that I come across e.g. new artists or recordings |
| I listen attentively to music for ___ per day |
| I often read or search the internet for things related to music |
| I spend a lot of my free time doing music related activities |
| I’m intrigued by musical styles I’m not familiar with and want to find out more |
| Music is kind of an addiction for me - I couldn’t live without it |
| **Perceptual Abilities (9 items, α=.873)** |
| I am able to judge whether someone is a good singer or not |
| I can compare and discuss differences between two performances or versions of the same piece of music |
| I can tell when people sing or play out of time with the beat |
| I can tell when people sing or play out of tune |
| I find it difficult to spot mistakes in a performance of a song even if I know the tune |
| I have trouble recognizing a familiar song when played in a different way or by a different performer |
| I usually know when I’m hearing a song for the first time |
| When I hear music I can usually identify its genre |
| When I sing I have no idea whether I’m in tune or not |
| **Musical Training (7 items, α=.903)** |
| I engaged in regular, daily practice of a musical instrument (including voice) for ___ years |
| I can play the following number of musical instruments (including voice) |
| I have never been complimented for my talents as a musical performer |
| At the peak of my interest, I practised ___ hours on my primary instrument (including voice) |
| I have had formal training in music theory for ___ years |
| During my life time I have had formal training on a musical instrument (including voice) for ___ years |
| I would not consider myself a musician |
| **Singing Abilities (7 items, α=.870)** |
| After hearing a new song two or three times, I can usually sing it by myself |
| I am able to hit the right notes when I sing along with a recording |
| I am not able to sing in harmony when somebody is singing a familiar tune |
| I can sing or play music from memory |
| I don’t like singing in public because I’m afraid that I would sing wrong notes |
| I only need to hear a new tune once and I can sing it back hours later |
| If somebody starts singing a song I don’t know, I can usually join in |
| **Emotions (6 items, α=.791)** |
| I am able to identify what is special about a given musical piece |
| I am able to talk about the emotions that a piece of music evokes in me |
| I often pick certain music to motivate or excite me |
| I sometimes choose music that can trigger shivers down my spine |
| Music can evoke my memories of past people and places |
| Pieces of music rarely evoke emotions for me |
| **General Musical Sophistication (18 items, α=.926)** |
| I would not consider myself a musician |
| I engaged in regular daily practice of a musical instrument (including voice) for ___ years |
| I have never been complimented for my talents as a musical performer |
| I can sing or play music from memory |
| At the peak of my interest I practised ___ hours on my primary instrument (including voice) |
| I am able to hit the right notes when I sing along with a recording |
| I spend a lot of my free time doing music related activities |
| Music is kind of an addiction for me - I couldn’t live without it |
| I don’t like singing in public because I’m afraid that I would sing wrong notes |
| When I sing I have no idea whether I’m in tune or not |
| After hearing a new song two or three times, I can usually sing it by myself |
| I can play the following number of musical instruments (including voice) |
| I only need to hear a new tune once and I can sing it back hours later |
| I often read or search the internet for things related to music |
| I am able to identify what is special about a given musical piece |
| I am not able to sing in harmony when somebody is singing a familiar tune |
| I enjoy writing about music for example on blogs and forums |
| I can compare and discuss differences between two performances or versions of the same piece of music |

Table S2

|  | Active Engagement | Perceptual Abilities | Musical Training | Singing Abilities | Emotions |
| --- | --- | --- | --- | --- | --- |
| Active Engagement | 1 |  |  |  |  |
| Perceptual Abilities | .637 | 1 |  |  |  |
| Musical Training | .521 | .657 | 1 |  |  |
| Singing Abilities | .562 | .806 | .719 | 1 |  |
| Emotions | .808 | .788 | .486 | .615 | 1 |

Table S3

*Data norms for subscales and general sophistication (sample n = 147,633)*

|  | Active Engagement | Perceptual Abilities | Musical Training | Singing Abilities | Emotions | General Musical Sophistication |
| --- | --- | --- | --- | --- | --- | --- |
| 1% | 15 | 29 | 7 | 9 | 21 | 32 |
| 2% | 18 | 32 | 7 | 12 | 23 | 37 |
| 3% | 20 | 35 | 7 | 13 | 24 | 41 |
| 4% | 22 | 36 | 7 | 15 | 25 | 43 |
| 5% | 23 | 37 | 8 | 16 | 26 | 46 |
| 6% | 24 | 38 | 8 | 17 | 27 | 48 |
| 7% | 25 | 39 | 9 | 18 | 27 | 50 |
| 8% | 26 | 39 | 9 | 19 | 28 | 51 |
| 9% | 27 | 40 | 10 | 19 | 28 | 53 |
| 10% | 28 | 41 | 10 | 20 | 28 | 54 |
| 11% | 28 | 41 | 11 | 21 | 29 | 55 |
| 12% | 29 | 41 | 11 | 21 | 29 | 56 |
| 13% | 29 | 42 | 11 | 22 | 29 | 57 |
| 14% | 30 | 42 | 12 | 22 | 29 | 58 |
| 15% | 30 | 43 | 12 | 23 | 30 | 59 |
| 16% | 31 | 43 | 13 | 23 | 30 | 60 |
| 17% | 31 | 43 | 13 | 23 | 30 | 61 |
| 18% | 32 | 43 | 14 | 24 | 30 | 62 |
| 19% | 32 | 44 | 14 | 24 | 30 | 63 |
| 20% | 33 | 44 | 14 | 25 | 31 | 64 |
| 21% | 33 | 44 | 15 | 25 | 31 | 64 |
| 22% | 34 | 44 | 15 | 25 | 31 | 65 |
| 23% | 34 | 45 | 16 | 26 | 31 | 66 |
| 24% | 34 | 45 | 16 | 26 | 31 | 67 |
| 25% | 35 | 45 | 17 | 26 | 31 | 67 |
| 26% | 35 | 45 | 17 | 26 | 32 | 68 |
| 27% | 35 | 45 | 18 | 27 | 32 | 69 |
| 28% | 36 | 46 | 18 | 27 | 32 | 69 |
| 29% | 36 | 46 | 19 | 27 | 32 | 70 |
| 30% | 36 | 46 | 19 | 28 | 32 | 71 |
| 31% | 37 | 46 | 20 | 28 | 32 | 71 |
| 32% | 37 | 46 | 20 | 28 | 33 | 72 |
| 33% | 37 | 47 | 21 | 28 | 33 | 73 |
| 34% | 38 | 47 | 21 | 29 | 33 | 73 |
| 35% | 38 | 47 | 21 | 29 | 33 | 74 |
| 36% | 38 | 47 | 22 | 29 | 33 | 75 |
| 37% | 39 | 48 | 22 | 29 | 33 | 75 |
| 38% | 39 | 48 | 23 | 29 | 33 | 76 |
| 39% | 39 | 48 | 23 | 30 | 34 | 76 |
| 40% | 39 | 48 | 23 | 30 | 34 | 77 |
| 41% | 40 | 48 | 24 | 30 | 34 | 77 |
| 42% | 40 | 49 | 24 | 30 | 34 | 78 |
| 43% | 40 | 49 | 25 | 31 | 34 | 79 |
| 44% | 41 | 49 | 25 | 31 | 34 | 79 |
| 45% | 41 | 49 | 25 | 31 | 34 | 80 |
| 46% | 41 | 49 | 26 | 31 | 34 | 80 |
| 47% | 41 | 50 | 26 | 31 | 35 | 81 |
| 48% | 42 | 50 | 26 | 32 | 35 | 81 |
| 49% | 42 | 50 | 27 | 32 | 35 | 82 |
| 50% | 42 | 50 | 27 | 32 | 35 | 82 |
| 51% | 42 | 51 | 28 | 32 | 35 | 83 |
| 52% | 43 | 51 | 28 | 33 | 35 | 84 |
| 53% | 43 | 51 | 28 | 33 | 35 | 84 |
| 54% | 43 | 51 | 29 | 33 | 36 | 85 |
| 55% | 43 | 51 | 29 | 33 | 36 | 85 |
| 56% | 44 | 52 | 29 | 33 | 36 | 86 |
| 57% | 44 | 52 | 30 | 34 | 36 | 86 |
| 58% | 44 | 52 | 30 | 34 | 36 | 87 |
| 59% | 45 | 52 | 30 | 34 | 36 | 87 |
| 60% | 45 | 53 | 31 | 34 | 36 | 88 |
| 61% | 45 | 53 | 31 | 34 | 37 | 89 |
| 62% | 45 | 53 | 31 | 35 | 37 | 89 |
| 63% | 46 | 53 | 32 | 35 | 37 | 90 |
| 64% | 46 | 53 | 32 | 35 | 37 | 90 |
| 65% | 46 | 54 | 32 | 35 | 37 | 91 |
| 66% | 46 | 54 | 33 | 36 | 37 | 91 |
| 67% | 47 | 54 | 33 | 36 | 37 | 92 |
| 68% | 47 | 54 | 33 | 36 | 38 | 93 |
| 69% | 47 | 55 | 34 | 36 | 38 | 93 |
| 70% | 48 | 55 | 34 | 37 | 38 | 94 |
| 71% | 48 | 55 | 34 | 37 | 38 | 94 |
| 72% | 48 | 55 | 35 | 37 | 38 | 95 |
| 73% | 48 | 56 | 35 | 37 | 38 | 96 |
| 74% | 49 | 56 | 35 | 38 | 38 | 96 |
| 75% | 49 | 56 | 36 | 38 | 39 | 97 |
| 76% | 49 | 56 | 36 | 38 | 39 | 98 |
| 77% | 50 | 57 | 36 | 38 | 39 | 98 |
| 78% | 50 | 57 | 37 | 39 | 39 | 99 |
| 79% | 50 | 57 | 37 | 39 | 39 | 100 |
| 80% | 51 | 58 | 38 | 39 | 39 | 100 |
| 81% | 51 | 58 | 38 | 40 | 40 | 101 |
| 82% | 51 | 58 | 38 | 40 | 40 | 102 |
| 83% | 52 | 58 | 39 | 40 | 40 | 103 |
| 84% | 52 | 59 | 39 | 41 | 40 | 103 |
| 85% | 53 | 59 | 40 | 41 | 40 | 104 |
| 86% | 53 | 59 | 40 | 41 | 40 | 105 |
| 87% | 53 | 60 | 40 | 42 | 40 | 106 |
| 88% | 54 | 60 | 41 | 42 | 41 | 107 |
| 89% | 54 | 60 | 41 | 43 | 41 | 107 |
| 90% | 55 | 61 | 42 | 43 | 41 | 108 |
| 91% | 55 | 61 | 42 | 43 | 41 | 109 |
| 92% | 56 | 61 | 43 | 44 | 42 | 110 |
| 93% | 56 | 62 | 43 | 44 | 42 | 111 |
| 94% | 57 | 62 | 44 | 45 | 42 | 113 |
| 95% | 57 | 62 | 44 | 45 | 42 | 114 |
| 96% | 58 | 63 | 45 | 46 | 42 | 115 |
| 97% | 59 | 63 | 46 | 47 | 42 | 117 |
| 98% | 60 | 63 | 46 | 48 | 42 | 118 |
| 99% | 61 | 63 | 47 | 49 | 42 | 121 |
| 100% | 63 | 63 | 49 | 49 | 42 | 126 |

Table S4

|  | GMS statistic | | GMS p-value | | MMT statistic | | MMT p-value | | BPT statistic | | BPT p-value | |
| --- | --- | --- | --- | --- | --- | --- | --- | --- | --- | --- | --- | --- |
| Musical Training | - | | - | | 58.181 | | .000 | | 67.020 | | .000 | |
| Active Engagement | - | | - | | 22.662 | | .000 | | 45.738 | | .000 | |
| Age | -36489 | | .000 | | 16.932 | | .000 | | -26.600 | | .000 | |
| Gender Female | -7.612 | | .000 | | -3.302 | | .055 | | -20.181 | | .000 | |
| Occupation |  | |  | |  | |  | |  | |  | |
| Accounting/Finance | -10.478 | | .000 | | -0.114 | | 1.000 | | -3.097 | | .106 | |
| Administration | -6.410 | | .000 | | -0.545 | | 1.000 | | -5.988 | | .000 | |
| Business development | -0.978 | | 1 | | -1.878 | | .965 | | -0.329 | | 1.000 | |
| Consultancy | -454 | | .000 | | 2.310 | | .687 | | 0.255 | | 1.000 | |
| Customer service | -1.62 | | .983 | | -5.816 | | .000 | | -1.332 | | 1.000 | |
| Education/Training | 11.446 | | .000 | | 9.927 | | .000 | | 0.772 | | 1.000 | |
| Engineering/R and D | -6762 | | .000 | | 2.052 | | .891 | | 1.968 | | .935 | |
| Executive/Senior management | -6410 | | .000 | | 1.656 | | .996 | | -1.953 | | .941 | |
| General management | -6.574 | | .000 | | -0.646 | | 1.000 | | -3.904 | | .005 | |
| Government/military | -6.828 | | .000 | | -1.475 | | 1.000 | | -2.951 | | .165 | |
| Healthcare | -6.786 | | .000 | | -1.431 | | 1.000 | | -4.181 | | .002 | |
| Homemaker | -6.598 | | .000 | | 0.326 | | 1.000 | | -4.818 | | .000 | |
| IT | -11.041 | | .000 | | 2.950 | | .167 | | 5.014 | | .000 | |
| Legal | -1.395 | | .999 | | 2.198 | | .786 | | -1.004 | | 1.000 | |
| Manufacturing/Operations | -5.121 | | .000 | | 0.358 | | 1.000 | | -0.288 | | 1.000 | |
| Media | 12.236 | | .000 | | 5.045 | | .000 | | 12.208 | | .000 | |
| Medical/Science | -6.369 | | .000 | | 3.137 | | .094 | | -1.551 | | .999 | |
| Music | 15.917 | | .000 | | 5.319 | | .000 | | 7.691 | | .000 | |
| Other | 15.360 | | .000 | | -9.881 | | .000 | | 0.192 | | 1.000 | |
| Personnel | -2.847 | | .166 | | -0.092 | | 1.000 | | -1.021 | | 1.000 | |
| Professional | -2.480 | | .398 | | 6.461 | | .000 | | -3.546 | | .022 | |
| Sales/Marketing/Advertising | 1.953 | | .855 | | -3.672 | | .014 | | 2.182 | | .800 | |
| Skilled Labour | -3.082 | | .083 | | -3.733 | | .011 | | -0.900 | | 1.000 | |
| Unemployed | 8.685 | | .000 | | -9.973 | | .000 | | 2.075 | | .880 | |
| Occupational Status |  | |  | |  | |  | |  | |  | |
| At university | 16.136 | | .000 | | -9.699 | | .000 | | 8.291 | | .000 | |
| Homemaker/full time parent | -7.412 | | .000 | | 0.617 | | 1.000 | | -6.186 | | .000 | |
| In full-time employment | -12.220 | | .000 | | 8.488 | | .000 | | 5.691 | | .000 | |
| In part-time employment | -4.809 | | .000 | | 3.185 | | .081 | | -4.497 | | .000 | |
| Retired | -18.807 | | .000 | | -0.246 | | 1.000 | | -23.316 | | .000 | |
| Self-employed | 10.549 | | .000 | | 12.375 | | .000 | | 9.730 | | .000 | |
| Still at school | 20.905 | | .000 | | -18.136 | | .000 | | -0.636 | | 1.000 | |
| Unemployed | -3.190 | | .058 | | -4.348 | | .001 | | -1.003 | | 1.000 | |
| Highest Educational Degree Obtained |  | |  | |  | |  | |  | |  | |
| A-levels | 4.810 | | .000 | | -5.956 | | .000 | | 3.149 | | .090 | |
| Completed GCSE/CSE/O-levels | -4.067 | | .002 | | -12.291 | | .000 | | -4.426 | | .001 | |
| Completed post-16 vocational course | -5.428 | | .000 | | -4.168 | | .002 | | -3.274 | | .059 | |
| Did not complete GCSE/CSE/O-levels | -7.063 | | .000 | | -11.372 | | .000 | | -10.390 | | .000 | |
| I am still in education | 1,808 | | .000 | | -14.937 | | .000 | | 3.344 | | .046 | |
| Postgraduate degree | -0.639 | | 1 | | 14.610 | | .000 | | 0.204 | | 1.000 | |
| Undergraduate degree or professional qualification | -9.442 | | .000 | | 14.837 | | .000 | | 1.863 | | .968 | |
| Highest Educational Degree Expected |  |  |  |  |  |  |  |  |  |  |  |  |
| A-levels |  |  | | -7.862 | | .000 | | -1.401 | | 1.000 | |  |
| GCSE/CSE/O-levels |  |  | | -6.887 | | .000 | | -4.881 | | .000 | |  |
| Not applicable |  |  | | 17.028 | | .000 | | -6.586 | | .000 | |  |
| Post-16 vocational course |  |  | | -2.820 | | .238 | | -0.467 | | 1.000 | |  |
| Postgraduate degree |  |  | | -4.081 | | .003 | | 8.343 | | .000 | |  |
| Undergraduate degree or professional qualification |  |  | | -13.114 | | .000 | | 2.739 | | .295 | |  |

*Footnote*. Values of the standardised test statistic and p-values from conditional inference tests derived from 50,000 permutations of the data for each test. P-values are adjusted by the single-step procedure for multiple testing on permutation statistics described in Westfall and Young (1993, algorithm 2.5 und formula 2.8). Column names designate scores of self-reported general musical sophistication (GMS), performance on the melodic memory test (MMT) and the beat perception test (BPT).
