# Supplementary material for: The Musicality of Non-Musicians: An Index for Assessing Musical Sophistication in the General Population
Source: PLoS One. 2014 Feb 26;9(2):e89642. doi: 10.1371/journal.pone.0089642 (PMC3935919; doi:10.1371/journal.pone.0089642)
Supplement: Textual Description S1 — (DOC) [file pone.0089642.s002.doc]

Supporting Textual Description S1

*The pilot survey*

The goals of the pilot survey were firstly to reduce the number of items in the pool of question items whilst ensuring that sufficient items remain to adequately index each latent dimension underlying distinct facets of musical sophistication. Secondly, we aimed to eliminate and adjust items such that for each dimension the range of levels of sophistication in the sample would be covered appropriately. For the pilot survey, items were randomised and implemented in a simple online survey tool. The survey was made publicly available for one week in November 2010 and participants were mainly recruited through a link from the Science section of the BBC website (http://www.bbc.co.uk/science/). The pilot was taken by 488 participants (306 females and 182 males) with most participants (58%) in the age categories of 25-34 (32%) and 35-44 (26%) years with lower percentages of younger and older participants. 37% of the participants had completed an undergraduate degree as their highest level of education while lower proportions of the sample had achieved a postgraduate degree (29%), were still in education (17%) or had only completed A-levels (10%). 83% of the sample indicated the UK as their current country of residency while the other 17% were almost evenly spread among 26 other countries. Similarly, the most common country where participants had spent their formative years of childhood and youth was the UK (80%), again with a wide and almost even spread across a large number of other countries. 89% indicated “White” as their ethnic background.

We first scanned the distributions of answers on all 111 items for any items where individual answer categories were extremely over-represented. We only identified one item where this was the case (72% of all answers allocated to the lowest category and 97% fell into the lowest three categories). This item (“I don't know what people mean when they say I have a tune going round my head”) was later removed from the item pool because of its low communality within the subsequent factor analyses. The Kaiser-Meyer-Olkin measure of sampling adequacy suggested that the correlational structure of the data was very suitable for a factor analysis (KMO = .931) and we therefore analysed the data with several factor analyses using principal axis factoring but varying the number of dimensions to be extracted (from 5 dimensions, the initial number of dimensions for item writing, to 13 dimensions, which was indicated as an upper bound by a parallel analysis (Horn, 1965) comparing simulated random data to the actual data from the pilot). From the 13-factor solution we inspected item communalities and removed all items that would not fit even with the maximal factor solution, i.e. we removed all items with a communality of 0.4 or lower. This removed 11 items from the item pool.

We then factor-analysed the data in a second iteration and reviewed each factor solution in terms of the number of items associated with each dimension, the eigenvalues of the factors, the communality of the items and the interpretability of solution after promax rotation. From this review we arrived at an 8-factor solution and removed items with communalities of 0.4 or less on the 8-factor solution. This resulted in the removal of a further 8 items. Factor 8 of this solution only comprised 2 very similar items asking for the building of playlists in iTunes or similar programs/devices. This appeared conceptually too narrow to warrant a separate dimension of musical sophistication and we therefore excluded these two items from the item pool as well. This left us with 89 items which were subjected again to a factor analysis leading to the adoption of a 7-factor solution.

Because we deemed 89 items still too many for a self-report inventory, and in order to optimise the psychometric properties of the seven individual dimensions, we then combined analytic approaches from item response theory (IRT) and classical test theory to decide on item inclusion. For each dimension we applied the graded response model (Samejima, 1969) to identify those items that had a low overall information content or covered a very similar region in ability space, as well as those items which would increase Cronbach's alpha considerably when excluded from their respective dimension. In most instances, IRT analysis and Cronbach's alpha coincided on the same items which were therefore excluded. Eventually, we arrived at 70 items on seven dimensions, where each dimension showed very good psychometric indicators (values of Cronbach's alpha ranging b etween .693 and .921). We factor-analysed the data from the final pool of 70 items using principal axis factoring and promax rotation. The 7-factor solution explained 53.6% of the total variance and inter-correlations between the seven factors were found to be low to moderate in magnitude (range: .071 to .617).

Horn, J. L. (1965). A rationale and test for the number of factors in factor analysis. Psychometrika, 30 (2), 179-185.

Samejima, F. (1969). Estimation of latent ability using a response pattern of graded scores. Richmond, VA: Psychometric Society.
